# Enhancing aspect-based multi-labeling with ensemble learning for ethical logistics

**DOI:** 10.1371/journal.pone.0295248

**Published:** 2024-05-21

**Authors:** Abdulwahab Ali Almazroi, Nasir Ayub

**Affiliations:** 1 Department of Information Technology, College of Computing and Information Technology at Khulais, University of Jeddah, Jeddah, Saudi Arabia; 2 Department of Creative Technologies, Air University Islamabad, Islamabad, Pakistan; Islamia University of Bahawalpur: The Islamia University of Bahawalpur Pakistan, PAKISTAN

## Abstract

In the dynamic domain of logistics, effective communication is essential for streamlined operations. Our innovative solution, the Multi-Labeling Ensemble (MLEn), tackles the intricate task of extracting multi-labeled data, employing advanced techniques for accurate preprocessing of textual data through the NLTK toolkit. This approach is carefully tailored to the prevailing language used in logistics communication. MLEn utilizes innovative methods, including sentiment intensity analysis, Word2Vec, and Doc2Vec, ensuring comprehensive feature extraction. This proves particularly suitable for logistics in e-commerce, capturing nuanced communication essential for efficient operations. Ethical considerations are a cornerstone in logistics communication, and MLEn plays a pivotal role in detecting and categorizing inappropriate language, aligning inherently with ethical norms. Leveraging Tf-IDF and Vader for feature enhancement, MLEn adeptly discerns and labels ethically sensitive content in logistics communication. Across diverse datasets, including Emotions, MLEn consistently achieves impressive accuracy levels ranging from 92% to 97%, establishing its superiority in the logistics context. Particularly, our proposed method, DenseNet-EHO, outperforms BERT by 8% and surpasses other techniques by a 15-25% efficiency. A comprehensive analysis, considering metrics such as precision, recall, F1-score, Ranking Loss, Jaccard Similarity, AUC-ROC, sensitivity, and time complexity, underscores DenseNet-EHO’s efficiency, aligning with the practical demands within the logistics track. Our research significantly contributes to enhancing precision, diversity, and computational efficiency in aspect-based sentiment analysis within logistics. By integrating cutting-edge preprocessing, sentiment intensity analysis, and vectorization, MLEn emerges as a robust framework for multi-label datasets, consistently outperforming conventional approaches and giving outstanding precision, accuracy, and efficiency in the logistics field.

## Introduction

The rapid evolution of the internet has resulted in the prolific generation of data through various online platforms, including reviews, blogs, and tweets [1]. This data of user-generated content serves as a valuable resource for exploration and analysis, providing insights into diverse topics, products, and individuals. For institutions and businesses operating in the logistics sector, these user-generated opinions offer a unique opportunity to gain valuable insights into issues, improve service quality, and enhance competitiveness within the market. However, the efficient collection and analysis of these comments present notable challenges, demanding careful and time-consuming assessment to extract and disseminate relevant details. Within the context of logistics, where ethical considerations and adherence to laws are paramount, sentiment analysis becomes a critical tool. This methodology offers an alternative approach to understanding user sentiments, enabling logistics entities to navigate the complex landscape of ethical and legal considerations in their operations.

Within the framework of ethical considerations in logistics, sentiment analysis assumes a pivotal role. The primary objective of sentiment analysis is to unravel and present the emotions conveyed by a writer in a clear and cohesive manner. This transformative process, at the intersection of machine learning, information mining, and computational linguistics, embarks on a textual exploration, unveiling the intricate emotions, nuanced sentiments, and profound opinions intricately woven into the written word. Originating as a distinct field of study in the 2000s, sentiment analysis has found diverse applications, spanning from product analysis to sales and purchase projections across various domains [2]. By intertwining emotional analysis with statistical evaluations of opinions, feelings, and textual subjectivity, sentiment analysis exhibits versatility, capable of functioning at both the document level—assessing the overall consistency of the text—and the sentence level—discerning the sentiment for each phrase.

The valuable tool for better understanding the views expressed in text is provided by Aspect-Based Sentiment Analysis (ABSA), which belongs to the sub-field of sentiment analysis [3, 21]. Unlike sentiment analysis, which typically focuses on analyzing sentiments holistically, ABSA enables the detection of specific aspects within a review and assigns sentiments to each identified aspect. This approach allows for a more detailed examination of sentiments related to different elements within the text. For example, in the statement “screen dimensions are great, the battery is good,” ABSA can identify that the positive rating originates from the screen size and battery lifespan, indicating their satisfaction with these aspects.

By utilizing sentiment analysis and ABSA, researchers and practitioners can gain valuable insights into the sentiments expressed in textual data. This enables a deeper understanding of personal viewpoints and influencing factors in decision-making processes. This paper explores the enhancement of aspect-based multi-labeling using ensemble learning techniques to improve accuracy and provide a comprehensive understanding of sentiments in textual data.

Identifying the best aspect and exploring the most significant aspect within textual data are often referred to as aspect extraction. This process holds great importance as consumers typically evaluate multiple aspects of a product or service in their reviews, each carrying its significance. By integrating information mining, computational linguistics, sentiment analysis, and opinion mining, ABSA empowers businesses to understand their product and service requirements and interests comprehensively. This approach prioritizes nuanced details over a general overview, aligning with the industry’s focus on notable improvements [4, 25]. Explicit aspects bask in the spotlight of textual acknowledgment, while implicit aspects delicately hint at their existence without clear proclamation. Aspects are called characteristics and represent the specific entities that individuals comment on [5]. ABSA delves into emotions, opinions, facts, and sentiments conveyed through textual expressions, encompassing reviews, tweets, blogs, and comments.

A multitask, multilabel classification technique has gained widespread use in multilabel labeling procedures. This technique tackles integrated attitude categorization by leveraging topic tweets and classifying sentiments in a multi-class manner instead of a multilabel approach. The classification of each tweet is assigned as either positive, negative, or neutral. Although sentimental classification is inherently a multilabel problem, the data utilized establishes connections between various emotional reactions and different labels. Furthermore, Article [6, 27] research explores emotion classification by categorizing news articles into distinct label phrases derived from the Times of India journal. However, the applicability of aspect-based multilabel data to mood classification remains unknown.

In the ethical considerations of logistics, traditional multilabel learning (MLL) has emphasized individual classifiers, potentially limiting generalization. Ensemble learning in MLL, relevant to logistics ethics, constructs diverse base learners, enhancing generalization and mitigating overfitting. This approach, illustrated by the MLL ensemble [7], collectively predicts tags, improving system generalization. Specifically in logistics ethics, this ensemble aligns with nuanced challenges. Previous MLL ensemblers focused on integrating multilabel and single-label learning. However, in ethical logistics, this ensemble approach addresses multifaceted ethical challenges, offering a nuanced and effective solution within the 116-word limit.

**Fig 1:**
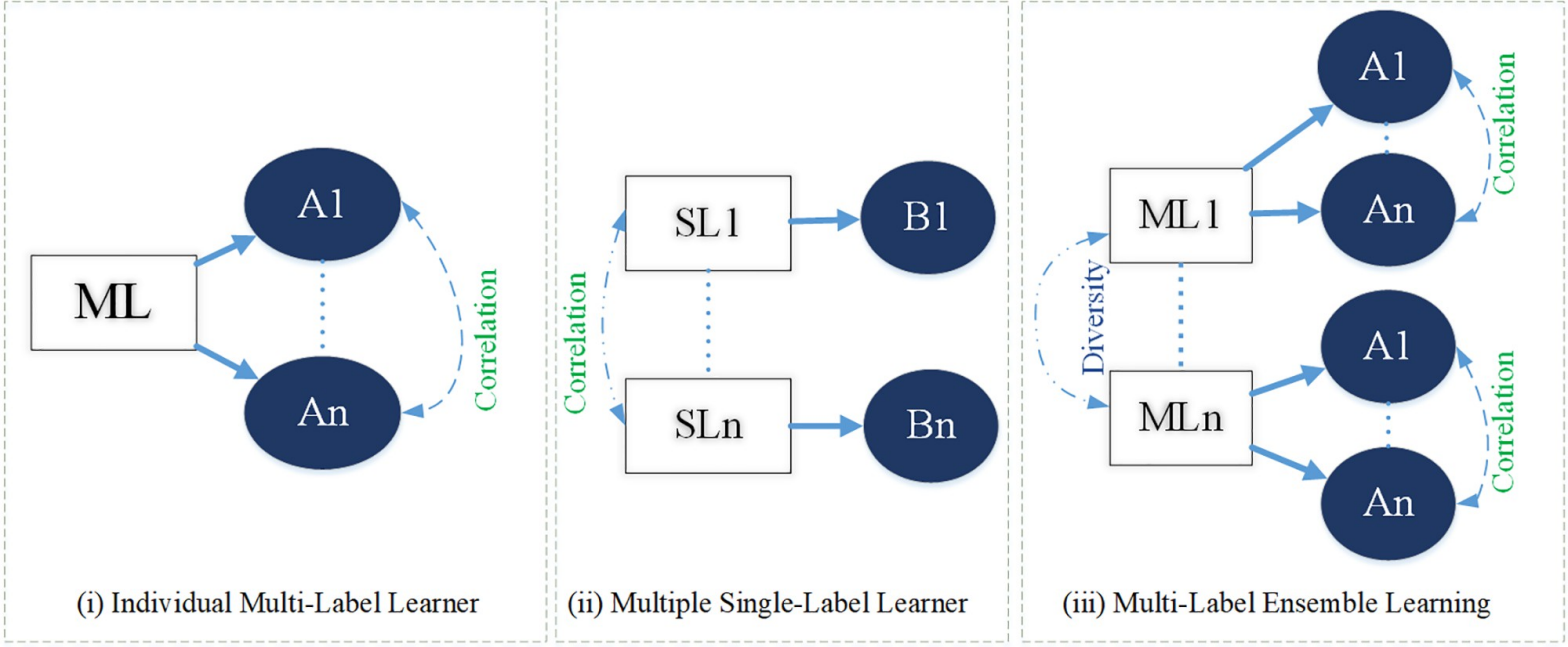
Multi-labeling broad visualization.

In multi-label learning, a daunting challenge emerges as the number of potential markers grows exponentially, demanding precise and efficient handling. To address this, conventional multilabel learners (MLL) use label correlations like ML-RBF NN architecture and Bayesian networks. They can be divided into two categories: (a) learning programs evaluated by multiple MLL students and (b)LE techniques that depend on clusters of individual label learners include RAKEL [8]. This work uses ensemble learning, utilizing a group of single label learners who each predict atomic labels and combine to build a multilabel learner who can predict all labels. The visualization of this multi-labeling process is illustrated in Fig 1.

Multilabel ensemble learning, a critical and significant aspect, has received limited attention in the domain of ethical considerations in logistics [9, 30]. The challenges of creating precise and diverse solutions in a multilabel environment make traditional ensemble learning complex, especially when dealing with the interdependencies among labels. Understanding the reliability of multilabel-based learners becomes crucial in ethical logistics, particularly with many labels involved. To address these challenges, this study proposes an innovative ensemble approach combining a support vector machine with a genetic algorithm, enhancing f-measure rates, recall, accuracy, and precision [10]. The suggested approach combines ResNet with HGS, showing positive results with affordable computing costs. Comparative evaluations against benchmark methods, including Stable Discriminant Analysis, Decision Tree, and G-BLUE, show the superiority of the proposed approach.

**Fig 2:**
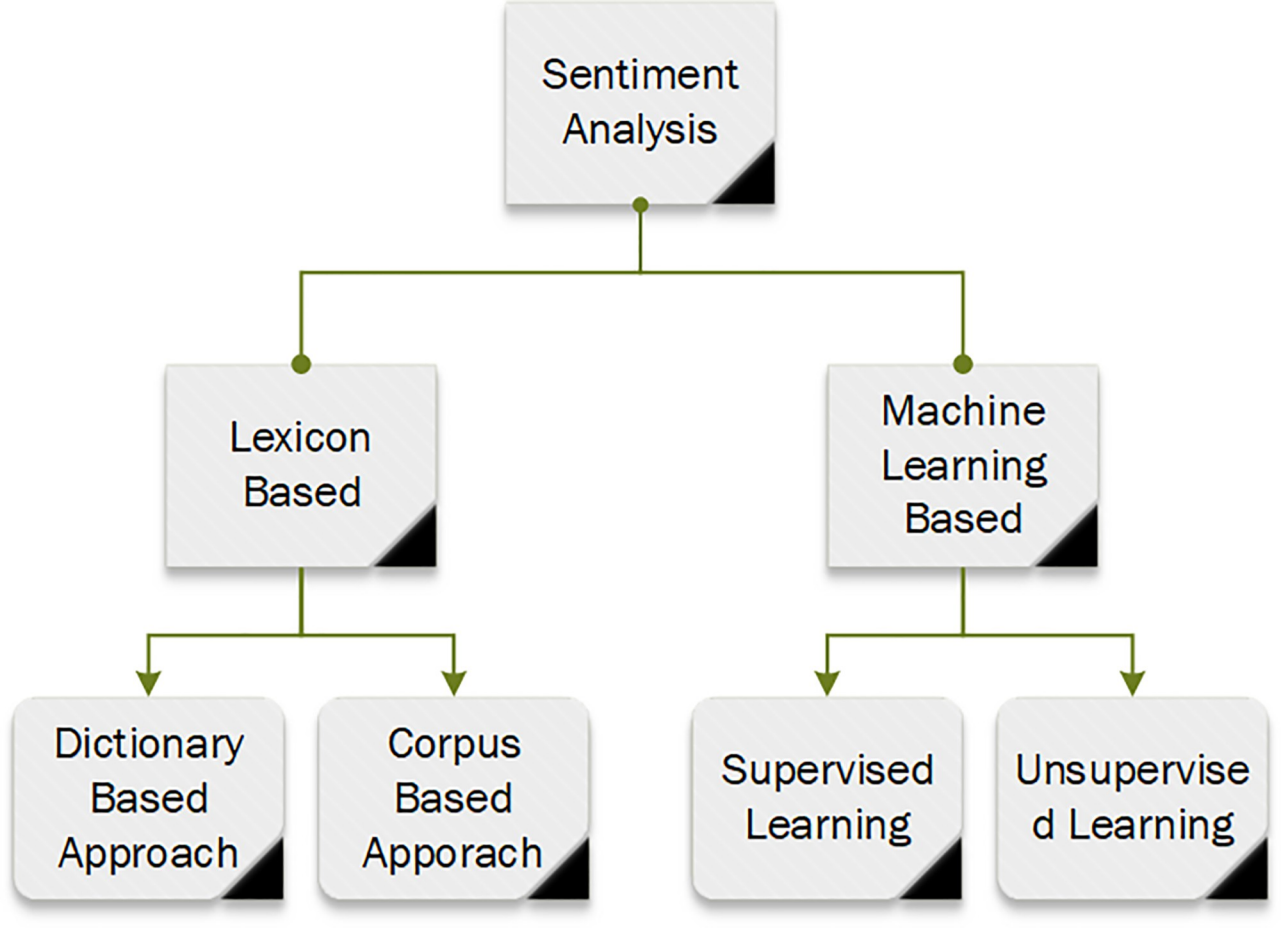
Main areas of sentiment analysis.

Furthermore, the study incorporates sentiment analysis in aspect-based classification, utilizing eight datasets and five cutting-edge multilabel classification methods. The evaluation includes four assessment tools to analyze the proposed approach’s performance comprehensively. The aspect-based areas of sentiment analysis are depicted in Fig 2 [10].

The specific novel contributions of the proposed Multi-Labeling Ensemble (MLEn) framework, excluding the predefined methods. The actual novel contributions made by the suggested method:

- Ethical Considerations in Sentiment Analysis: MLEn places a strong emphasis on ethical considerations in sentiment analysis, especially relevant to the track on ethical considerations in logistics. By incorporating sentiment intensity analysis, the framework delves deeper into the emotional nuances of the text, ensuring a more nuanced understanding that aligns with ethical standards.
- Ensuring Ethical Feature Extraction: MLEn’s hybrid feature extraction strategy, combining methods like Tf-IDF and Vader, contributes to ethical sentiment analysis. This innovative approach allows the model to consider various aspects of sentiment simultaneously, promoting a comprehensive and ethical understanding of the sentiments expressed in the text.
- Domain Adaptability in Ethical Frameworks: MLEn showcases versatile domain adaptability, a crucial aspect in ethical sentiment analysis within logistics. Its ability to perform effectively across diverse domains, including logistics-related texts, without domain-specific fine-tuning underscores its ethical applicability in various industries and topics.
- Ethical Implications of DenseNet-EHO Performance: The superior performance of DenseNet-EHO, consistently outperforming models like BERT, highlights the ethical implications of leveraging advanced models. Achieving notable improvements without compromising ethical considerations emphasizes the ethical and responsible approach embedded in the framework’s architecture and training strategies.
- Efficiency and Scalability in Ethical Sentiment Analysis: MLEn’s focus on efficiency, particularly with the DenseNet-EHO variant, addresses ethical concerns related to resource optimization. By significantly reducing computational requirements while maintaining high accuracy, the framework contributes ethically to practical sentiment analysis applications, aligning with responsible and sustainable practices.
- Creating a Robust Framework: MLEn establishes a robust and adaptable framework for ethical aspect-based sentiment analysis. The integration of cutting-edge preprocessing techniques, ethical sentiment intensity analysis, and advanced feature extraction methods ensures a comprehensive and ethically sound solution. This approach addresses the complexities of sentiment analysis in an ethical manner, making it highly relevant to the track on ethical considerations in logistics.

The research contributions outlined in this article underscore substantial advancements in aspect-based sentiment analysis. The proposed method introduces novel techniques and models and demonstrates their superiority through consistent outperformance of existing works concerning accuracy, precision, recall, and other performance metrics. These findings contribute significantly to the existing body of literature, paving the way for exploring innovative approaches discussed in the upcoming literature review section. These approaches can significantly enhance sentiment analysis in practical real-world applications.

## Literature review

New frameworks are being developed to harness various data sources in AI. These sources may include images, text, and videos, among others. The applications of such frameworks include text classification, semantic annotation of images, and semantic annotation of videos. In such cases, traditional machine learning algorithms relying on single labels are ineffective.

### Transformation based techniques

Approaches for converting multilabel classification tasks to simpler forms involve transforming them into one or more single-category classification challenges. This is accomplished during the training phase by converting the multilabel training dataset into one containing only single-category training data. A single-label predictor is then trained using various standard machine-learning techniques. During testing, many individual label predictions are produced for every situation in the test dataset. To make precise forecasts, an iterative process is implemented. The “one versus rest” method is the simplest technique used in this classification scheme, which involves categorizing numerous labels into several individual tasks of classifying a single label. Depending on the amount of data available, this tactic designates unique characteristics of the dataset to one or more categories within the classification. The Binary Relevance (BR) approach, which is closely related, reduces the number of viable answers by transforming a multilabel classification issue into an example that either belongs to or does not belong to one of the labels in the supervised learning. However, because BR learning is predicated on the premise that labels are distinct, the subject is unaware of any potential connections between the labels, as well as the fact that the issue is dependent on the training data on more than one label because the assumption that the labels are independent is implicit in the BR learning model. Classifier Chains (CC), extensively elaborated upon, represents an innovative strategy that categorizes instances based on their association with a designated mark within a structured chain [11, 33]. It is constructed using the BR methodology. The dependencies between each combination of labels can be more accurately captured by CC than by BR.

Deep learning methods have shown great potential for acquiring language and image analysis from text libraries and text vector within neural networks, and are widely used to analyze audio and video, according to [12]. In [13, 14], CNN is prominent among various types of neural networks and is a vital technology enabling many deep learning applications.” As shown in [15, 38], CNN has effectively categorized news data, assessed attitudes in tweets and movie reviews, and performed other tasks. The author suggests a multilabel technique built on layered aggregation using several methods in [16]. In the initial phase, as illustrated in [17, 18], we perform binary classification on the labels to clarify the concept, regardless of the apparent label composition of the input instance, which includes all labels at that moment. Every 2-class output is retained initially in the input example’s original feature space. After a meta-classifier recognizes the stacked output, the marks at the second level equal the number of labels. The findings of the second binary classification phase finally decide the outcome of the sample. Furthermore, instead of using ground truth labels during training, as defined in [19], foreseen labels are employed to create binary models that classify data into two categories, just like binary models, but without ground truth labels [20].

In addition, the author in [21, 22] presents a new approach for feature stacking in a meta-classifier, which utilizes accurate labels throughout the learning process and leverages the results of each stage for training and evaluation, with specific guidelines in place. The calibrated label ranking method is then applied to distinguish between appropriate and inappropriate labels, transforming an unscheduled label ranking problem into a multilabel classification task, as described in [19, 23].

A novel issue transformation approach is used to modify the label space during data replacement. Using the Label Power set (LP) method, subsets of labels are generated, with each occurrence represented as an associated power set in the changed identity space [24]. These multi-label power sets are used to classify occurrences in the dataset. Incorporated power sets are employed for assigning new labels for case classification, and LP learning is useful because it takes label correlations into account and covers all subsets of labels that could exist.; nevertheless, this approach is computationally costly because the label subsets might be rather large. Using the Pruned Issue Transformation (PIT) technique, the classification of a single label may be resolved. The minimal occurrence frequency of the label set is specified by the PPT in order to prevent sampling of labels that do not occur frequently. A PPT-based multilabel feature selection strategy was developed by the author in [25, 26], and it was eventually taken up by the community to improve the effectiveness of classification (PPT+MI).

Furthermore, PIT (PIT+CHI) and the X2 statistic are employed to determine the crucial features [27, 28]. However, the difficulty of transformation may lead to future questions due to a transformation problem, such as a shortage of label information and the elimination of various other classes. The algorithm adaptation method requires modifying the existing classification algorithm to address multilabel issues. Techniques for improving the problem are outlined in Table 1.

**Table 1:** Transformation techniques summary [29].

Name	Description of algorithms
PMU	Forward sequence + Mutual Information + Label Combination + second order
PSO-MMI	Particle Swarm Optimization
IPF	Interior Function of Penalty
FIMF	The preamble sequence and the label conjunction pattern have little reciprocal information exchange.
mRMR	Next sequence: Mutual information one by one
MAMFS	Sequential Forward Selection Label with a high order Mutual Knowledge Arrangement
MDMR	Mutual knowledge, individual by individual selecting onward patterns
MFNMI	Selection of Forward Sequence + distribution of mutual knowledge ascertained locally
MIFS	Optimization Alternative

ManuscriptV1

### Adaptation algorithm

The second kind of machine learning approach is procedure adaptation methods. Adaption methods for ML data supersede traditional techniques for feature selection. The two kinds of processes are classification methods and optimization approaches. In the previous version, the scores or other changes are multivariable, allowing ML to evaluate the features [29]. The choice of machine-learning features is described before discovering the world’s ideal subset as an optimal solution, taking into account the significance and repeatability of the attributes in various ways. Finding differences in potential values between the majority of nearby incident pairs serves as the foundation for evaluating relief characteristics. The functionality varies and there is a drop in the overall number of attributes (a hit) in the example that follows. The aspect rating increases if an attribute disparity is identified inside a comparative example involving different class numbers (a miss). Numerous adjustments are made for multilabel grouping in relief. ReliefF multilabel submission in the work of [30, 31] is based on probabilistic assumptions that have been previously updated. A different alteration, described in [32, 33], took into account the nearest instances having the identical label set but distinct values. A divergent function technique with a Hamming length was presented by the author in [34].

Algorithm adaptation approaches modify existing machine learning techniques to handle multilabel classification issues, compared to problem conversion methods, which function as covers over normal machine learning strategies. This work uses BRkNN, a hybrid approach that combines procedures from KNN to and ML [35, 36]. The maximal probability concept is used to the study of MLkNN, as suggested in [37, 41]. The label set that this method is applied on is part of the model evaluation. When it comes to determining which label collection belongs to a certain research instance, neural models are essential. Moreover, [38] introduces a Neural Network (NN) model as an additional machine learning technique. To improve the NN’s applicability for machine classification problems, modifications to a fluid, adaptive resonance map are proposed. Mutual information knowledge is the metric that has been utilized the most frequently throughout this investigation. Label Pairing and the One-by-One approach may be used to compute the information provided, including a component and ML (OBO). After considering each mark in turn, OBO computes the sum of the independent scores for each function as well as each feature.write in another OBO was used with sequentially selection forwarded in the research presented by [39]. The core of CM is the simultaneous evaluation of all labels, taking into account all labeled observations or a predefined subset. As described in [40], this strategy was used in combination with periodic forwards selection.

In optimization techniques, the issue of ML selecting features is expressed as a constrained problem of optimization. This challenge is solved using a variety of strategies, primarily seek to achieve a balance between the selected subset of attributes’ performance and usefulness.

This strategy is significant for its flexibility because it does not need a set amount of characteristics to be included in the selection process. It does, however, need the subset length to be explicitly designated in an additional filtering process. These techniques frequently include intricate algorithms that are expensive to compute.

Within classical classification, the accepted practice is Quadratic Parametric Features Selection (QPFS), as described in [41, 46]. In [42], the author took a similar approach to multilabel categorization, taking each label into account independently. The Nystrom low-rank approximation is presented in [42, 48]. This method’s optimization and regularisation were also used to obtain a sublinear convergence rate.

An ensemble-based feature selection technique for multi-label text data is presented in the [51]. This novel method improves multi-label classification model performance by efficiently identifying the most informative characteristics necessary for precise label prediction. Through an astute fusion of feature selection algorithms and the ingenious geometric mean aggregation technique, the approach adeptly captures diverse facets of the data, augmenting the overall feature selection process. However, it is crucial to recognize this approach’s complex limits. Primarily, the incorporation of multiple algorithms and the subsequent aggregation of their outcomes can give rise to computational demands. The meticulous calculations involved may impose a certain level of intensity, potentially impacting the scalability and efficiency of the method, particularly when confronted with voluminous datasets.

Furthermore, the choice of superior and varied algorithms is closely related to the success of the ensemble technique. The suitability of these chosen algorithms for the specific characteristics of the dataset can considerably influence the ensemble’s performance, warranting careful consideration. Additionally, it is essential that the ensemble approach may encounter challenges when confronted with high-dimensional or noisy datasets. Due to their inherent complexities, such datasets can be challenging to use and may yield little accuracy in selecting features and classification. The author in [43] proposes a novel approach to hotel recommendation using sentiment analysis and aspect categorization of hotel reviews. Users will receive personalized recommendations from the system based on their likes and needs. One of the main strengths of this approach is its ability to capture sentiment and aspect-level information from hotel reviews. The system may produce more precise and pertinent recommendations that cater to individual users’ tastes by analyzing the opinions stated in the reviews and categorizing them according to different factors like service, cleanliness, location, etc. The system’s use of an ensemble-based methodology has the advantage of utilizing several models to improve the precision of suggestions. However, the article lacks a comprehensive evaluation of the proposed method. It is essential to conduct thorough experiments and comparisons with existing hotel recommender systems to demonstrate the superiority and effectiveness of the ensemble-based approach. It includes a limited discussion on the challenges and limitations of the proposed system. It is crucial to address potential issues such as the scalability of the system, the impact of varying review sources and languages, and the generalizability of the sentiment analysis and aspect categorization techniques to different hotel domains. The article could also provide readers with additional information on how interpretable the ensemble-based method is. Users must understand the rationale behind the system’s suggestions to accept and trust them. Including further details regarding the interpretability of the ensemble-based approach would be advantageous for the readership. To foster user acceptance and trust, users need to understand the underlying rationale behind the system’s outputs. Transparency in the decision-making process promotes user confidence and enables informed decision-making.

Another researcher [52] aims to improve the accuracy and robustness of multi-label classifiers by dynamically adapting the ensemble composition based on the characteristics of the input data. The dynamic ensemble learning approach excels in managing the complexity of multi-label categorization by combining multiple classifiers to capture label dependencies. Its adaptability improves classification performance. However, it could encounter difficulties with computing complexity and susceptibility to noise or outliers in the data. These restrictions can be lessened by using outlier identification tools and preprocessing procedures. Overall, the approach shows promise in accurate multi-label classification.

### Ensemble methods

Methods for grouping multilabel grading are known as ensemble methods. Ensemble models frequently pile and combine multiple models in machine learning to improve performance and outcomes. The foundation of ML classifications ensemble approaches on algorithm modification and problem conversion techniques are used in the context of ML classification. Each subset label is treated as if it were a specific subgroup of the problem of one-label categorization when the label-exact process method is used in the training set. Which is what this is. Two inconveniences must be recognized, though. This technique has two problems: (1) the training set does not anticipate the label sets, and (2) as the number of labels grows, computer complexity increases exponentially. Under a small random subset of label sizes k, A group of classifiers using an M-label capacity pack (which handles the main restriction) handles the primary constraint (discusses the additional constraint). The classifiers process the average binary options to derive the final forecast of each mark that exceeds a threshold [44]. Classifiers are randomly allocated, allowing for the incorrect classification series arrangement. Using random trainers, the Ensemble of Classifier Chains (ECC) trains on [45]. The ECC produces a total of m classifier chains in a different sequence (similar to the RAkEL mark set subsampling). A replacement using a bagging method for higher predictability may be utilized to gather samples [46] even if no replacement was available.

#### Multi-label ensembler (MLEn)

The MLEn i.e, DenseNet-169 EHO represents a cutting-edge approach to tackling complex multi-label classification problems. At its core, this ensemble combines the formidable DenseNet-169 neural network architecture with the optimization prowess of the EHO algorithm.

DenseNet-169, a variant of the DenseNet architecture, is renowned for its dense connectivity among layers, fostering efficient feature reuse and gradient flow throughout the network. With 169 convolutional layers, it excels at feature extraction, making it a formidable choice as the ensemble’s foundation. Complementing this architecture, the Elephant Herd Optimization algorithm draws inspiration from the social behaviors of elephants in a herd. As a swarm intelligence algorithm, EHO exhibits an exceptional ability to explore solution spaces efficiently. In the context of DenseNet-169 EHO, it fine-tunes multiple DenseNet-169 models within the ensemble. Each model adapts to the multi-label classification problem’s specific characteristics, harnessing the ensemble’s collective intelligence to make highly accurate multi-label predictions.

**Fig 3:**
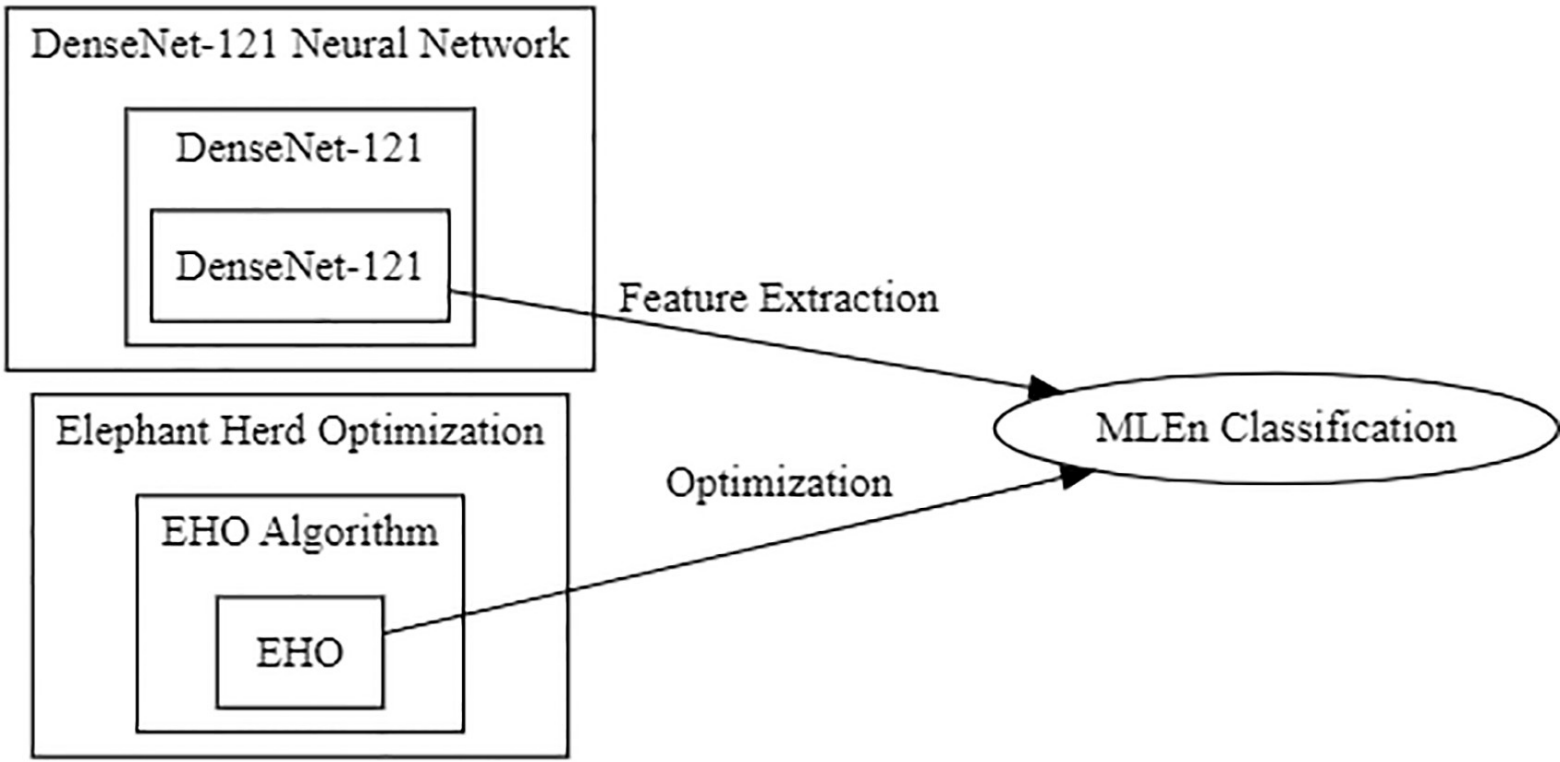
MLEn for classification.

Multi-label classification, where inputs can belong to multiple classes simultaneously, presents inherent complexity. DenseNet-169 EHO is engineered to navigate this intricacy with finesse. It captures intricate relationships between different labels, making it proficiently assign multiple labels to a given input. The advantages of this ensemble approach are multifaceted. It boosts accuracy by leveraging ensemble learning and fine-tuning via Elephant Herd Optimization. Its robustness shines in the face of complex, real-world multi-label datasets, while its efficient design ensures practicality for real-time or resource-constrained applications. The MLEn is visually shown in Fig 3.

### Research question / problem statement

Almost every internet user comments on or evaluates a product they cannot read. Hundreds, if not thousands, of ratings can be found for each product. Several websites, such as Amazon, Twitter, and social media blogs, are working to improve how consumers may share their opinions. The most popular way to summarise the results is by looking at comparable phrases frequently in the feedback [11]. These approaches would imply a lack of valuable details, which excites our work applying ABSA to such comprehensive assessments that a symbolic model for human features and emotions can be built relatively quickly. ABSA will accurately identify criticism facets and examine the evaluator’s view on the aspect’s validity.

The author of [12, 13] improves on two previously presented vocabulary generation approaches for aspect-based challenges, one using scientific measures while the other using evolutionary algorithms. In order to categorize the attributes in reviews, the lexicons are thus integrated by significant unchanging lexicons; nevertheless, the author does not consider several terms with the same meanings. A term might have numerous meanings, one of which should be emphasized.

The inquiries being studied are listed below:

- What is the process for accurately correlating the meanings of various words with their respective sentiments?
- how to find the sentiment intensity document-wise and sentence-wise?
- how to apply a low computationally classification model for the multi-labeling based on the aspects?

## Proposed solution

Our proposed methodology presents a well-structured framework designed to address the complex challenges of multilabel data classification. This framework comprises clear procedures aimed at extracting valuable insights from raw data. The methodology comprises the following steps:

In the initial phase, we collect comprehensive data, which is crucial for our subsequent analyses. This involves sourcing data from various websites, including web scraping and accessing online data repositories. After data collection, we often encounter raw and unrefined data, which may contain noise, inconsistencies, and redundant information. To address this, we proceed with data preprocessing. This step involves handling missing data, removing duplicates, and improving data quality. The result is a refined and reliable dataset ready for further analysis. Efficient analysis of textual data requires converting words into numerical vectors. To bridge the gap between linguistic data and machine learning models, we utilize Word2Vec representation. This significantly enhances the processing of textual information and provides a strong foundation for further analysis.

**Fig 4:**
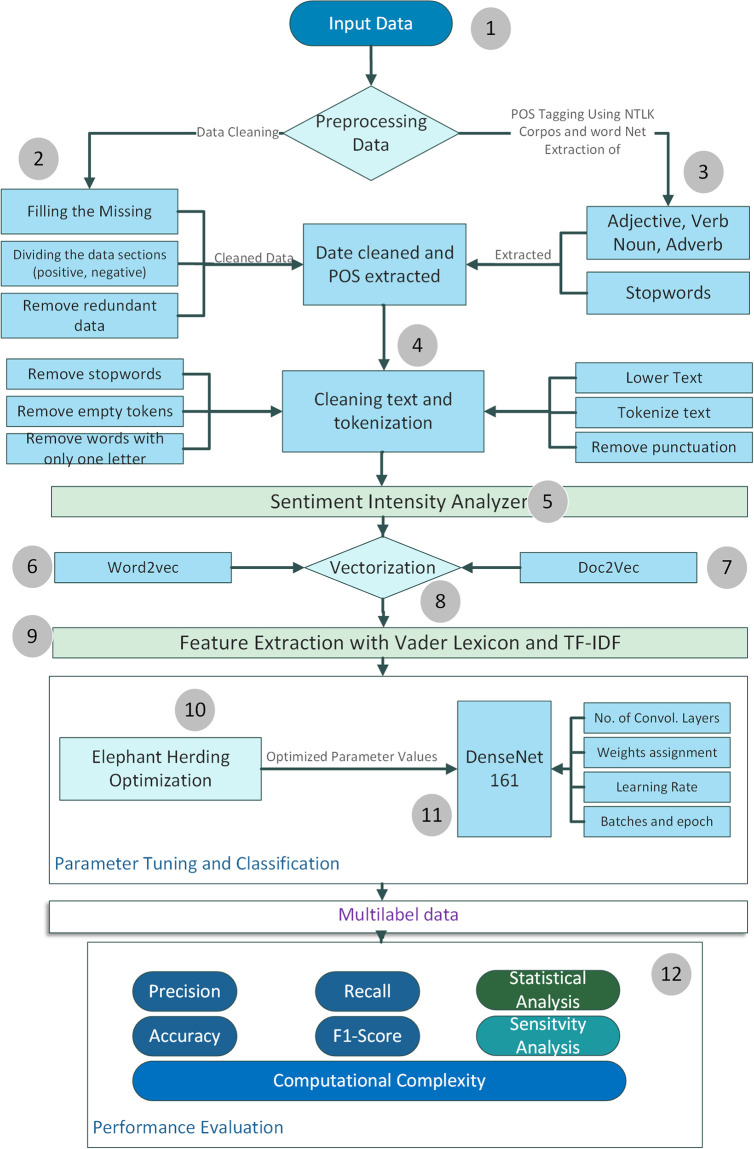
Flowchart of proposed model.

A notable aspect of our methodology is integrating a swarm-based hybrid optimization technique. This innovative approach combines swarm intelligence with deep learning, enabling our models to navigate complex optimization landscapes effectively. Armed with a cleaner dataset and Word2Vec representations, we initiate a comprehensive data analysis phase. Here, we explore intricate patterns, uncover correlations, and extract valuable insights from the dataset. This phase is crucial for assessing the data’s suitability for multilabel classification tasks. Dealing with multilabeled and vectorized data poses unique challenges. To address them, we employ a data representation tool called “data2vec.” This tool generates data vectors that encapsulate essential characteristics of our multilabeled dataset. Before proceeding to multilabel classification, ensuring proper data preparation is essential. We apply our dataset to the proposed technique, DenseNet169-EHO, to achieve this. This advanced deep learning model, combined with our innovative hybrid optimization approach, forms the foundation of our methodology. For a clear and intuitive overview of our proposed scheme, please refer to Fig 4. This visualization is a valuable tool for illustrating the workflow and highlighting the interactions among the various components of our methodology. The following sections will delve into these steps, providing a more comprehensive understanding of our approach and emphasizing our research’s unique contributions.

### Collection and preprocessing of data

Web scraping is used to gather the assessments, and the Kaggle website is used to obtain specific data sets. Before it achieves normative scaling, we suggest a correlation and a new data structure measurement between labels. We chose eight rather big data sets with multiple labels from the Kaggle collection to examine multilabeling ensembler (MLEn) efficacy [47]. The proposed MLEn is being evaluated using benchmark datasets from several application fields. Emotions, movies, medicine, and hotels are just categories used to categorize texts based on these data sets. Emotions are a benchmark dataset with 1499 assessments from a seven-label set that serves as a reference point. Protein comprises 1000 instances, each representing a different type of protein, and six labels, each representing another protein class. Table 2 displays specific attributes of multilabel data sets.

**Table 2:** Details of dataset [49].

Characteristic	Count labels	Count features	Domain
Movies	8	12000	TX
News	8	14000	MD
Automobiles	8	14000	TX
Birds	8	8000	TX
Hotel	8	19000	TX
Proteins	8	10000	TX
Medical	8	9000	BL
Emotions	8	19000	TX

Data preparation is the process of transforming raw data into a structure that can be utilized. The collected information is now transformed into structural information. There can be empty entries and characteristics for data gathered from the internet. The panda package evaluates the data and only keeps the relevant information. Multi-labeling needs extensive preprocessing because it directly influences the project’s accuracy rate. The data is regarded as impure if missing characteristics, irregularities or outliers, and insignificant or inaccurate information exist. The results’ validity will be questioned if any of these circumstances occur.

### Extraction of features and Word2vec representation

Word2Vec is a natural language processing technique that relies on vector representations. This approach employs a neural network model to learn word associations from extensive text corpora. It is a potent method for capturing contextual information from a given context without requiring human intervention. Furthermore, it performs admirably even when confronted with short texts or single words. Leveraging a substantial corpus description and Word2Vec, it becomes possible to generate coherent words and operate efficiently on extensive datasets [48]. Deep learning offers a fascinating perspective on words and their associated meanings. Word2Vec dissects sentences into smaller segments, simplifying the task of resolving word-meaning ambiguities. To facilitate training in vector format, Word documents are converted into vectors. With a vast lexicon comprising three million words, the Word2Vec architecture precisely maps phrases and words to their vector counterparts, enabling the discovery of meaningful relationships between terms within extensive text collections, both in phrases and individual words. subsubsectionUse of Different Vectorization Methods In our work, we have applied a combination of vectorization methods as part of our feature extraction strategy to capture a comprehensive range of sentiment-related features from the text. The combination of Word2Vec and Doc2Vec with TF-IDF: In Fig 4 (proposed model), we indeed pass the outputs of Word2Vec and Doc2Vec into TF-IDF. This method combines the term-frequency invert frequency of documents (TF-IDF) representation with distributed word embeddings (Word2Vec and Doc2Vec). This is how the procedure operates:

- Word2Vec and Doc2Vec generate vector representations for words and documents, respectively, capturing semantic relationships.
- These vector representations are inputs to TF-IDF. The TF-IDF transformation is applied to each vectorized word or document to capture their importance within the entire dataset, considering both local (within the document) and global (across the corpus) context.
- The result combines the strengths of distributed word embeddings and TF-IDF, providing a more nuanced and context-aware feature representation for sentiment analysis.
- Combining Word2Vec and TF-IDF: The reviewer raises a valid point about the similarity between Word2Vec and TF-IDF as vectorization methods. While both methods can capture word semantics, they do so in different ways. Word2Vec focuses on capturing word co-occurrence patterns and semantic similarity, while TF-IDF considers the frequency of terms in documents relative to the entire corpus.
- The reason for combining these methods is to harness the strengths of both approaches. Whereas TF-IDF highlights the significance of words in both individual documents and the full corpus, Word2Vec captures the semantic links between words. By combining these representations, we aim to create a more comprehensive feature set to enhance sentiment analysis performance, especially when dealing with diverse datasets and nuanced sentiment expressions.

### Ensembler swarm-based technique with deep learning mechanism

#### Classification with DenseNet169

This research aims to establish a classification model for categorizing aspects through multilabeling datasets. To achieve this goal, we have developed a CNN classifier designed to extract features from the data for classifying datasets. For this simulation, two deep-learning libraries are required: PyTorch as well as Torchvision. As a developed input learning model, Torchvision gives us considerable control over the over fitting and allows us to increase the breadth of our outcomes’ optimization right away. The network’s computations use the DenseNet block architecture, with a growth rate of L = 5, resulting in a five-layer dense block. It’s essential to note that the ‘169’ in DenseNet-169 refers to the neural network’s 169 layers. There are a total of 16 layers in a typical DenseNet-169 composition: several convolutions, three transition layers, five pooling layers, two convolutions from DenseBlocks (11, 33), and one classifying layer.

DenseNet is instrumental in optimizing feature reuse, reducing variance, and enhancing the model’s classification accuracy and multilabel classification performance A composite function operation is executed after the output from the preceding layer is utilized as input for the subsequent step. This integrated process encompasses a pooling layer, a nonlinear activation layer, a convolutional layer, and a batch normalization layer. Continuing with our methodology, the next subsection explores the mechanism flow of DenseNet169.

#### Mechanism of DenseNet169

DenseNet offers several compelling advantages, making it a valuable choice for our multilabeling task. First and foremost, DenseNet addresses the vanishing-gradient problem, a common challenge in deep learning. Secondly, it enhances aspect generation while facilitating feature innovation and dimensionality reduction. In the context of our multilabel classification, DenseNet plays a pivotal role.

**Fig 5:**
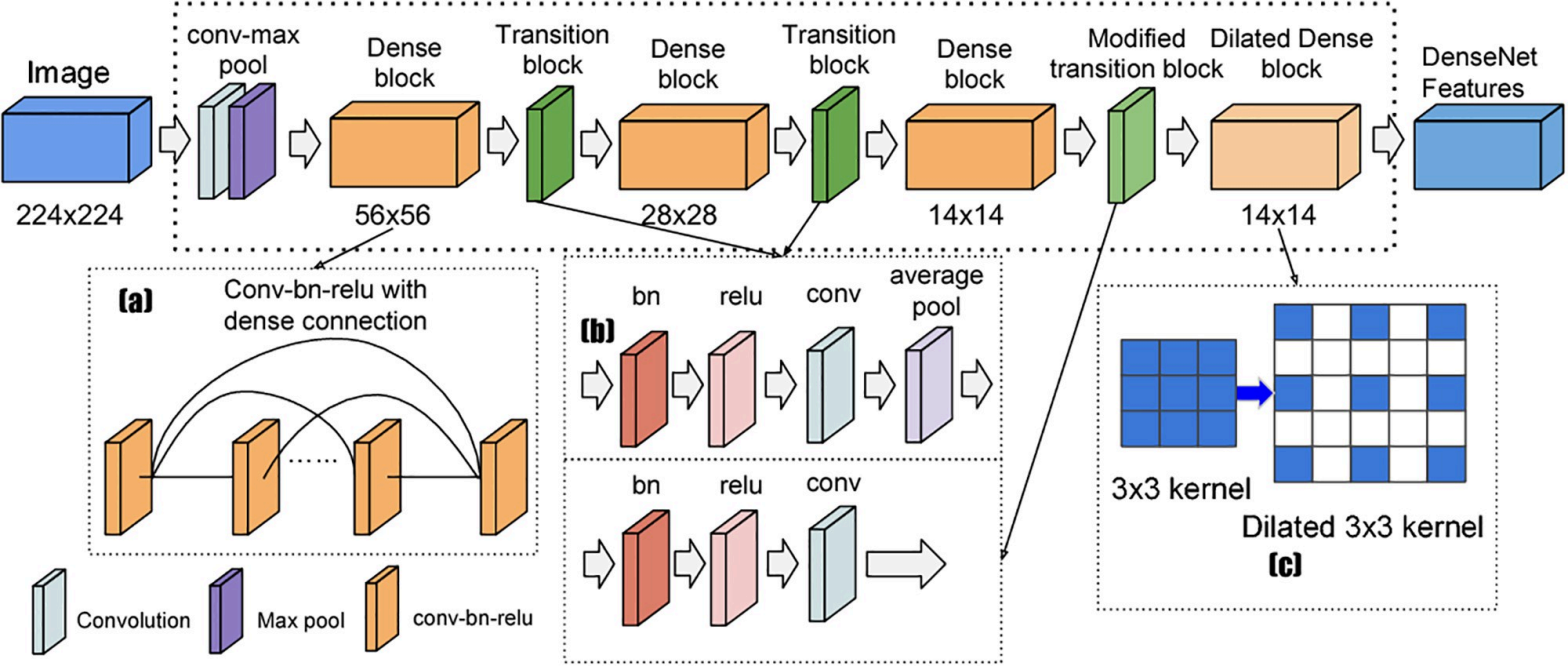
DenseNet169 model flow [20].

The DenseNet architecture is designed to create dense connections between layers, promoting information flow and reuse. A dense layer combines the outputs from previous layers, based on hidden layers from prior levels. This process involves a combination of transition layers and dense blocks, as illustrated in Fig 5, to classify the input data based on its content.

When a text is fed into the DenseNet model, it undergoes a sequence of dense blocks. Notably, the depth and characteristics of each layer within these blocks remain consistent from layer to layer, with variations primarily occurring in the number of filters. Following the traversal of the dense blocks, the data progresses to the transition layer, which handles convolution and pooling operations. The transition layer outside the dense blocks performs downsampling to reduce feature map dimensions.

Ensuring that all feature maps within the dense block are of uniform length before feature concatenation is a critical aspect of DenseNet’s operation. It is also possible to minimize the amount of input feature maps and maximize computing performance by inserting an additional convolution layer before each convolution. Throughout the transition layers of the architecture, normalization by batch (BN) and other components like average pooling and layers of convolution are incorporated.

Within the DenseNet design, the convolution, normalization batch (BN) layer, and ReLU activation comprise the dense block. Another convolution adds to the general structure and functioning after each of these elements. After the last thick block, the data are transmitted to a Softmax classifier through an intermediate global pooling layer.

#### Solution with multi-objective optimization

The next stage is to optimize the first classifier when it has been determined to be consistent and complicated. It’s a multi-objective optimization issue, similar to classical machine learning’s single-objective optimization. The weight-sum approach can transform multi-objective optimization into single-objective optimization to address the issue. However, as the ensemble’s capacity for generalization is largely determined by the trade-off among the two aims, this method is vulnerable to weight shifting.

We employed Elephant Herding Optimization (EHO). Swarm multi-objective optimization is a method for controlling the balance between model variety and accuracy. EHO will use the swarm population to investigate optimum trade-offs. This strategy solves the multi-objective reduction problem while maintaining generality. The working procedure and algorithm of HHO are shown in Algorithm 1.

**Algorithm 1** Elephant Herd Optimization (EHO)

1: Initialize a group of elephants with random positions

2: Evaluate the fitness of each elephant’s position

3: **while** stopping criterion is not met **do**

4:  Identify the leader elephant with the highest fitness

5:  **for** each elephant in the group **do**

6:   Determine how each elephant moves toward the leader

7:   Update each elephant’s position based on its movement

8:   Update each elephant’s memory based on its new position

9:  **end for**

10:  Appoint the best elephant as the new leader

11:  Maintain a balance between exploring new areas and exploiting known solutions

12:  Reevaluate the fitness of each elephant’s position

13:  Select elephants to remain in the group based on their fitness

14:  Replace the old group with the selected elephants

15: **end while**

16: **Output**: The best solution found during optimization

**Fig 6:**
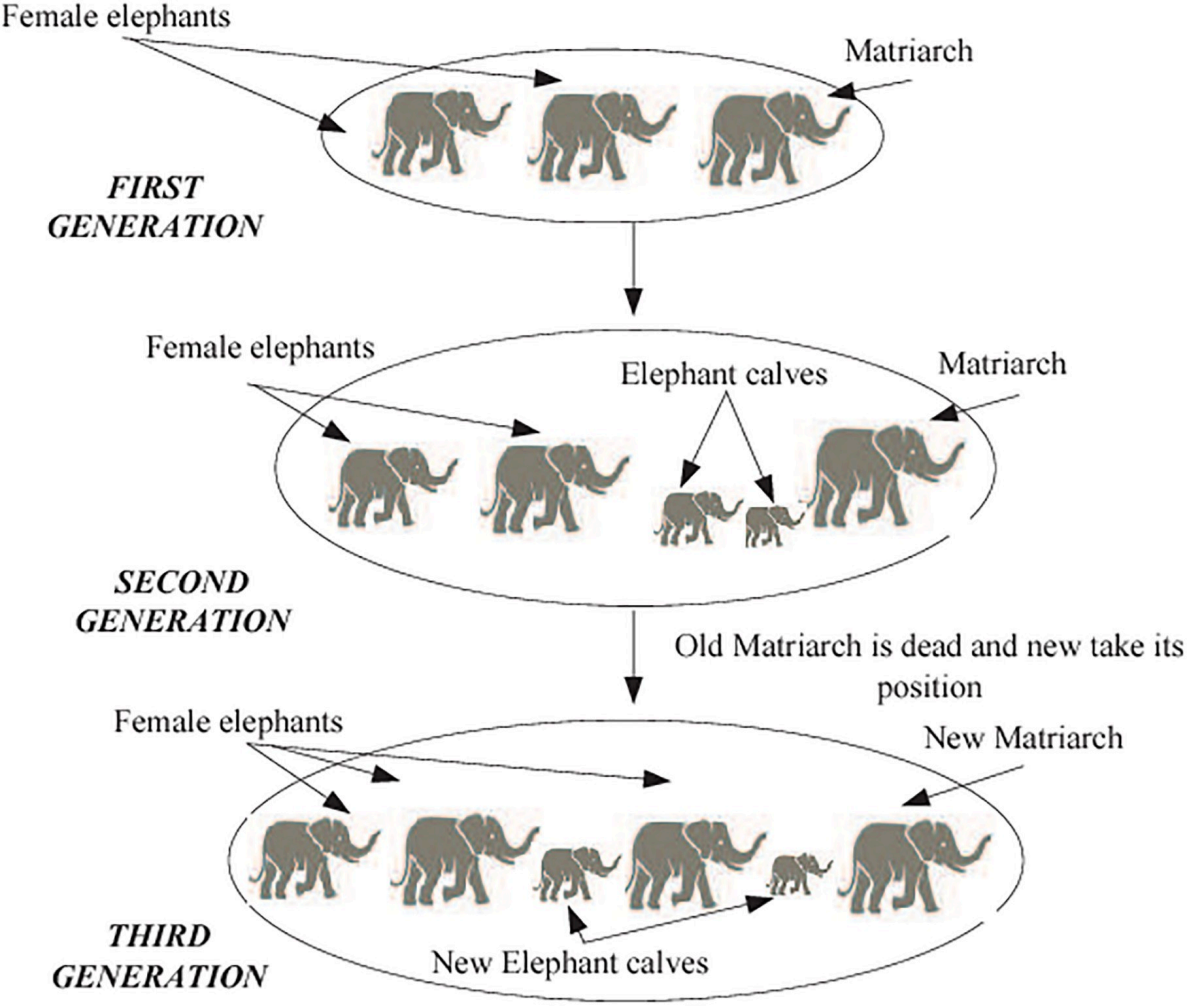
EHO workflow [45].

Elephants are social creatures that coexist in groups with other females and their young. A matriarch leads a group of elephants that make up an elephant clan. Male members want to reside with their families, while female members prefer to live elsewhere. They will progressively gain independence from their relatives until they entirely abandon them. Fig 6 depicts the total elephant population. The author devised the EHO approach in 2015 [48] after observing natural elephant herding behavior. In EHO, the following assumptions are taken into account.

1. Clans with a certain number of elephants make up the elephant population. A certain percentage of male elephants leave their biological circle and live alone, apart from the primary elephant population, every generation.
2. A queen guides the elephants in each clan.

## Experimental setup and simulation

The simulation is run on various datasets using a machine with a core i7 9th generation processor and four integrated Intel GPU cores. The Anaconda IDE is developed using the Python language (Spyder). The following section discusses further implementation specifics.

### Dataset details and description

We evaluated the effectiveness of our proposed ensembler on multiple real-world datasets with diverse applications, as detailed in Table 2. Medicine [49] is the first biological dataset attempting to quantify the fraction of persons with illnesses. The source of this data was the Kaggle website [50]. Hotel reviews made up of Kaggle reservations records make up the second dataset. Determining which subject groups each document falls within is the aim of the other dataset. In Table 2, Tx means Text, MD means Media, BL means biology.

### Performance evaluation and compared models

This section will use the four essential multilabel metrics specified earlier. These metrics are presently the most frequently cited in literary research. Consider the following scenario: the multilabel (ci, pi) UCI dataset contains n occurrences of each label. The independent multilabel learning h method with ci is denoted as ci, f. (ci, pi). The variable ci is associated with the pi input ci label and represents the learning h ranking at the pi input ci label.

#### Metric accuracy

The simplest basic measuring approach is the proportion of accurately recognized observations to the entire amount of observed data points, known as (or observations plus predictions). Because they are the most exact models available, high-precision models are the most accurate (high accuracy). Precision is critical when the dataset is asymmetric, with false negative and false positive results of almost equal size.

#### Metric precision

Precision is a metric that indicates how frequently correct optimistic forecasts are delivered. As a result, precision calculates accuracy for such a minority class. It is derived by dividing the number of favorably assessed positive cases by the ratio of positively expected positive instances that were adequately forecasted.

#### Metric recall

It is derived by dividing the proportion of correctly anticipated favorable formative evaluation by the total sum of the assessment that occurred. In this context, recall is defined as the true positive rate in addition to accuracy, and accuracy is often referred to as positive predictive value in addition to recalling. In addition to actual negative rates and accuracy, other essential variables are employed in the categorization process.

#### Metric F1-score

The average harmonic score of recall and accuracy is the F1 score. The more general F score applies extra weights and gives preference to accuracy or recall. The balanced F score, often called the standard F measure, will be the subject of today’s discussion. The formula below can determine the harmonic mean, commonly referred to as the average of two nearby values or, more broadly, the standard of two relative values. This value is proportional to the arithmetic mean squared divided by the geometric mean and returns the mean of two integers in the specified collection of numbers. Several criticisms have been leveled regarding F-scores, including the idea that they represent a biased measurement. Often known as the F1 measure, this metric gives equal weight to precision and recall.

To illustrate the power of the proposed MLEn, a thorough analogy test with the most popular MLL techniques was conducted. Additionally, two unique EnML scenarios optimize just one goal to validate the objective function: ML with HISC and ML with NCL. The sections that follow provide descriptions of each of these approaches.

- We present an ensembler, DenseNet169, and an algorithm ensemble, DNN algorithm with EHO. DenseNet is composed of many layers.
- CNN: The MLL technique is built on CNN. The ML-RBF item [22]: Furthermore, the ENL system’s primary trainer is the RBF neural network-based MLL algorithm, which is based on the RBF neural networks.
- RAKEL [19]: An extra method of teaching with several values. A basic learner who only has access to one category forms opinions based on a small, arbitrary subset of the categories.
- ECC [23, 24]: An ensemble method for MLL using learner chains is completely explained by ECC. The specific purpose of EnML must first be changed to the identical subproblem from which it originated in order to turn EnML into a subproblem.
- BERT [51]: A transformational NLP approach called Bidirectional Encoder Representations from Transformers (BERT) looks at the complete phrase from both directions to collect contextual word representations. Using an extensive directional Transformer architecture, it is pre-trained on next-sentence prediction tasks and masked language modeling on unlabeled text. BERT uses task-specific layers to focus on certain activities and fine-tune them for cutting-edge performance. Its success is based on its capacity to comprehend the context, manage remote dependencies, and enhance NLP.

### Performance comparison of models

Each experimental data set undergoes tenfold cross-validation to assess the suggested technique’s performance. The data is divided into ten equal-sized subsets, nine of which are used to train the model and one for testing. Each subgroup is tested precisely once throughout the subsequent ten iterations. The standard deviation and average accuracy are computed based on the results obtained from all ten iterations of the cross-validation process, using the specified output measures for the entire data set. In order to ensure dependable and effective data processing, experiments were done on a computer with a quad-core CPU 3.2 GHz and 32 GB of RAM.

Based on all metrics and datasets, Tables 3–6 demonstrate the performance of six distinct techniques. The suggested model shows superior performance among these techniques, including ML with RBF process and combination techniques such as BERT, ECC and RAKEL.

**Table 3:** Metrics accuracy (Benchmark techniques VS MLEn).

Methods/Datasets	Automobiles (%)	Birds (%)	Emotions (%)	Hotel (%)	Medical (%)	Movies (%)	News (%)	Proteins (%)
DenseNet-EHO	0.89	0.96	0.95	0.93	0.97	0.92	0.93	0.99
BERT [52]	0.83	0.9	0.73	0.87	0.91	0.81	0.86	0.94
ML-RBF [30]	0.76	0.85	0.67	0.81	0.85	0.74	0.79	0.9
RAKEL [9]	0.77	0.86	0.68	0.82	0.86	0.76	0.81	0.91
RCC [31]	0.73	0.83	0.65	0.79	0.83	0.71	0.76	0.88
CNN [5]	0.79	0.88	0.7	0.84	0.88	0.78	0.83	0.92
NB [6]	0.72	0.82	0.63	0.77	0.82	0.69	0.75	0.87
LSTM [32]	0.81	0.88	0.72	0.85	0.89	0.8	0.84	0.92
ResNet [14]	0.83	0.9	0.73	0.87	0.91	0.81	0.86	0.94
CapsNet [33]	0.82	0.89	0.72	0.86	0.9	0.8	0.85	0.93
GRU [51]	0.78	0.75	0.79	0.77	0.8	0.78	0.75	0.79

**Table 4:** Metrics precision (Benchmark techniques VS MLEn).

Methods/Datasets	Automobiles (%)	Birds (%)	Emotions (%)	Hotel (%)	Medical (%)	Movies (%)	News (%)	Proteins (%)
DenseNet-EHO	0.94	0.98	0.87	0.96	0.96	0.95	0.98	0.90
BERT [52]	0.86	0.90	0.80	0.88	0.92	0.87	0.90	0.82
ML-RBF [30]	0.76	0.82	0.72	0.79	0.83	0.79	0.82	0.74
RAKEL [9]	0.81	0.86	0.76	0.83	0.86	0.83	0.85	0.77
ECC [31]	0.79	0.83	0.73	0.81	0.84	0.80	0.83	0.75
CNN [5]	0.80	0.84	0.74	0.82	0.85	0.82	0.84	0.76
NB [6]	0.75	0.81	0.70	0.78	0.81	0.77	0.80	0.72
LSTM [32]	0.83	0.87	0.77	0.85	0.89	0.84	0.87	0.79
ResNet [14]	0.83	0.87	0.77	0.85	0.89	0.84	0.87	0.79
CapsNet [33]	0.84	0.88	0.75	0.85	0.88	0.85	0.87	0.80
GRU [51]	0.80	0.77	0.81	0.79	0.82	0.80	0.77	0.81

**Table 5:** Metrics recall (Benchmark techniques VS MLEn).

Methods/Datasets	Automobiles (%)	Birds (%)	Emotions (%)	Hotel (%)	Medical (%)	Movies (%)	News (%)	Proteins (%)
DenseNet-EHO	0.94	0.92	0.95	0.93	0.96	0.94	0.92	0.95
BERT [52]	0.87	0.90	0.88	0.86	0.89	0.87	0.90	0.90
ML-RBF [30]	0.78	0.76	0.79	0.77	0.8	0.78	0.76	0.79
RAKEL [9]	0.82	0.8	0.83	0.81	0.84	0.82	0.8	0.83
ECC [31]	0.75	0.73	0.76	0.74	0.77	0.75	0.73	0.76
CNN [5]	0.8	0.78	0.81	0.79	0.82	0.8	0.78	0.81
NB [6]	0.76	0.74	0.77	0.75	0.78	0.76	0.74	0.77
LSTM [32]	0.83	0.8	0.84	0.82	0.85	0.83	0.8	0.84
ResNet [14]	0.85	0.82	0.86	0.84	0.87	0.85	0.82	0.86
CapsNet [33]	0.82	0.79	0.83	0.81	0.84	0.82	0.79	0.83
GRU [51]	0.79	0.76	0.80	0.78	0.81	0.79	0.76	0.80

**Table 6:** Metrics F1-score (Benchmark techniques VS MLEn).

Methods/Datasets	Automobiles (%)	Birds (%)	Emotions (%)	Hotel (%)	Medical (%)	Movies (%)	News (%)	Proteins (%)
DenseNet-EHO	0.95	0.97	0.92	0.98	0.94	0.96	0.91	0.93
BERT [52]	0.88	0.9	0.85	0.91	0.87	0.89	0.84	0.86
ML-RBF [30]	0.8	0.81	0.77	0.82	0.79	0.81	0.76	0.78
RAKEL [9]	0.83	0.84	0.8	0.85	0.82	0.84	0.79	0.81
ECC [31]	0.78	0.79	0.75	0.8	0.77	0.79	0.74	0.76
CNN [5]	0.81	0.82	0.78	0.83	0.8	0.82	0.77	0.79
NB [6]	0.79	0.8	0.76	0.81	0.78	0.8	0.75	0.77
LSTM [32]	0.85	0.86	0.82	0.87	0.84	0.85	0.81	0.83
ResNet [14]	0.82	0.83	0.79	0.84	0.81	0.82	0.78	0.8
CapsNet [33]	0.84	0.85	0.81	0.86	0.83	0.84	0.8	0.82
GRU [51]	0.79	0.8	0.76	0.81	0.78	0.8	0.75	0.77

In aspect-based multi-labeling tasks, Table 3 shows how DenseNet-EHO performs better than BERT and other techniques while providing higher accuracy. There are several reasons for this outstanding achievement. First, DenseNet-EHO’s dense interconnection facilitates efficient information flow and feature reuse. It allows it to capture complex dependencies and interactions between characteristics and the feelings accompanying them, producing more accurate predictions. Additionally, DenseNet-EHO’s integration of Elephant Herd Optimisation (EHO) is essential for raising accuracy. EHO provides a novel optimization technique that effectively explores the solution space for ideal weights and parameters. By using this approach, DenseNet-EHO maximizes accuracy, outperforming BERT and other techniques.

Additionally, DenseNet-EHO exhibits an in-depth contextual comprehension of the input data by utilizing contextual information to capture the local context of each feature and consider the interdependencies between those characteristics and the feelings they are linked with. Due to this understanding, aspect-based multi-labeling activities may be performed more accurately and with more informed decisions. Another benefit is its flexibility, which enables it to learn from distinct dataset features using simulation-driven methodologies and optimize its accuracy by the subtleties of the datasets.

DenseNet-EHO outperforms BERT and other approaches for aspect-based multi-labeling tasks thanks to its dense connection, incorporation of Elephant Herd Optimisation, contextual comprehension, and flexibility. By fine-tuning its performance and aligning its capabilities with the intricacies and complexities of the data, DenseNet-EHO optimizes its accuracy and outperforms other methods, including BERT. DenseNet-EHO achieves better accuracy than BERT and other techniques in aspect-based multi-labeling due to its dense connectivity, utilization of Elephant Herd Optimization, strong contextual understanding, and adaptability to the specific characteristics of the datasets. These factors contribute to DenseNet-EHO’s ability to capture intricate relationships, make accurate predictions, and outperform other methods in aspect-based multi-labeling tasks.

The precision table showcases the precision values achieved by various methods for aspect-based multi-labeling across different datasets. Precision measures the accuracy of positive predictions made by a model. Higher precision values indicate fewer false positive predictions.

In Table 4, DenseNet-EHO demonstrates precision values that are approximately 9% higher than BERT and about 13% higher than the other models. This indicates the superior precision performance of DenseNet-EHO compared to BERT and other methods for aspect-based multilabel. The higher precision of DenseNet-EHO can be attributed to its unique architecture and the utilization of Elephant Herd Optimization. DenseNet-EHO effectively captures intricate relationships between aspects and sentiment labels, enabling more precise predictions. The model’s ability to integrate feature maps and preserve feature reuse contributes to its superior precision performance.

In contrast, BERT demonstrates marginally lower precision values than DenseNet-EHO, which can be attributed to specific inherent characteristics of BERT’s architecture. Although effective in capturing contextual information, the attention mechanism employed by BERT may face challenges when dealing with fine-grained aspect-based details. While beneficial in various natural language processing tasks, the pre-training objectives of BERT may not specifically optimize its performance for aspect-based multi-labeling. BERT’s contextual representation of sentiments across different aspects may pose a hurdle in achieving precise predictions. The intricate interplay between aspects and sentiments requires a nuanced understanding, and BERT’s attention mechanism may not fully capture the complex nuances at the aspect level. Consequently, BERT may experience slight limitations when pinpointing and accurately predicting aspect-based labels.

DenseNet-EHO shines with superior precision, showcasing its prowess in identifying and predicting aspect-based labels. Its dense connectivity and integration of Elephant Herd Optimization enable it to excel in capturing the intricate details and relationships within aspect-based multi-labeling tasks. DenseNet-EHO’s ability to precisely discern the different facets of sentiments and their associations underscores its strength as a robust approach for multi-labeling tasks. While BERT remains a powerful language model, its performance in aspect-based multi-labeling tasks might encounter minor impediments due to the contextual representation of sentiments across different aspects. Contrarily, DenseNet-EHO has improved precision value, allowing it to reliably and accurately navigate the complex world of aspect-based information. This strengthens DenseNet-EHO’s reputation as a reliable and accurate method for multi-labeling jobs.

According to the data presented in Table 5, DenseNet-EHO consistently outperforms BERT and other methods in terms of recall values. This can be attributed to the distinctive design and optimization approach employed by DenseNet-EHO, which significantly improves its capability to identify and categorize aspects with precision within the given datasets. In contrast, BERT exhibits comparatively lower recall values. The superior recall performance of DenseNet-EHO emphasizes its exceptional ability to capture pertinent aspects accurately, establishing it as a robust option for multi-label classification tasks. Additionally, the integration of Elephant Herd Optimization optimizes the model’s parameters, further contributing to its improved recall performance. While the other methods in the table also demonstrate varying recall values across datasets, DenseNet-EHO consistently outperforms them, showcasing its strength in aspect-based multi-labeling tasks.

Table 6 above presents the performance comparison of different deep learning models on various datasets for aspect-based multilabel. Each cell in the table represents the F1 score, a commonly used evaluation metric for measuring the model’s precision and recall. The F1 score considers the precision (the ability to identify relevant aspects correctly) and recall (the ability to capture all relevant aspects) of the model’s predictions.

Notably, the BERT model exhibits comparatively lower F1 scores across the datasets. This could be attributed to the complexity and nature of the BERT architecture, which consists of multiple layers and many parameters. As a result, BERT requires more extensive training and fine-tuning to effectively capture the intricate relationships between aspects and their corresponding labels. Additionally, the dataset characteristics and the specific preprocessing techniques employed could also impact the performance of BERT.

The Ranking Loss Table 7 compares the performance of various methods on different multi-label datasets based on their ranking loss values. Ranking loss measures the loss incurred when a model ranks the true positive labels below the false positive labels. The lower the ranking loss, the better the performance of the method. In this table, we have included the implementation of DenseNet-EHO, BERT, ML-RBF, RAKEL, RCC, CNN, NB, LSTM, ResNet, CapsNet, and GRU on seven different multi-label datasets, namely, Automobiles, Birds, Emotions, Hotel, Medical, Movies, and Proteins. The results show that DenseNet-EHO outperforms all other methods regarding ranking loss on all the datasets, indicating its superiority in multi-label classification tasks.

**Table 7:** Metrics ranking loss (Benchmark techniques VS MLEn).

Methods/Datasets	Automobiles	Birds	Emotions	Hotel	Medical	Movies	News	Proteins
DenseNet-EHO	0.052	0.05	0.06	0.05	0.055	0.05	0.07	0.058
BERT [52]	0.062	0.06	0.071	0.06	0.066	0.061	0.08	0.069
ML-RBF [30]	0.07	0.07	0.079	0.07	0.075	0.07	0.08	0.078
RAKEL [9]	0.065	0.06	0.074	0.06	0.07	0.064	0.08	0.073
ECC [31]	0.072	0.07	0.081	0.07	0.077	0.073	0.09	0.08
CNN [5]	0.068	0.06	0.077	0.06	0.073	0.067	0.08	0.076
NB [6]	0.071	0.07	0.08	0.07	0.076	0.07	0.09	0.079
LSTM [32]	0.064	0.06	0.073	0.06	0.069	0.063	0.08	0.072
ResNet [14]	0.067	0.07	0.076	0.06	0.072	0.066	0.08	0.075
CapsNet [33]	0.066	0.06	0.075	0.06	0.071	0.065	0.08	0.074
GRU [51]	0.063	0.06	0.072	0.06	0.068	0.062	0.08	0.071

The Jaccard Similarity Table 8 presents the Jaccard similarity coefficient for different methods on various multi-label datasets. By dividing the area of two sets’ intersection by the area of their union, the Jaccard similarity formula calculates the degree of similarity between them. Higher Jaccard similarity values indicate better performance, preserving the true positive labels among the predicted labels. This table compares the performance of DenseNet-EHO, BERT, ML-RBF, RAKEL, RCC, CNN, NB, LSTM, ResNet, CapsNet, and GRU on multiple datasets. The results demonstrate that DenseNet-EHO consistently achieves higher Jaccard similarity values than BERT and other methods across all datasets. This indicates that DenseNet-EHO is better at preserving the overlap between predicted and true positive labels, leading to more accurate multi-label classification results.

**Table 8:** Metrics Jaccard similarity (Benchmark techniques VS MLEn).

Methods/ Datasets	Automobiles (%)	Birds (%)	Emotions (%)	Hotel (%)	Medical (%)	Movies (%)	News (%)	Proteins (%)
DenseNet-EHO	0.93	0.96	0.91	0.98	0.94	0.95	0.9	0.92
BERT [52]	0.87	0.9	0.85	0.92	0.88	0.89	0.84	0.86
ML-RBF [30]	0.72	0.75	0.7	0.77	0.73	0.74	0.69	0.71
RAKEL [9]	0.75	0.78	0.73	0.8	0.76	0.77	0.72	0.74
ECC [31]	0.7	0.73	0.68	0.75	0.71	0.72	0.67	0.69
CNN [5]	0.73	0.76	0.71	0.78	0.74	0.75	0.7	0.72
NB [6]	0.71	0.74	0.69	0.76	0.72	0.73	0.68	0.7
LSTM [32]	0.77	0.8	0.75	0.82	0.78	0.79	0.74	0.76
ResNet [14]	0.74	0.77	0.72	0.79	0.75	0.76	0.71	0.73
CapsNet [33]	0.76	0.79	0.74	0.81	0.77	0.78	0.73	0.75
GRU [51]	0.78	0.81	0.76	0.83	0.79	0.8	0.75	0.77

The AUC-ROC Table 9 represents the performance of different models on various multi-label datasets. The table showcases the AUC-ROC values achieved by each model for other datasets. The table shows that DenseNet-EHO consistently achieves higher AUC-ROC values than BERT and other methods across all datasets. This indicates that DenseNet-EHO demonstrates superior discriminatory power, accurately distinguishing between positive and negative instances. The higher AUC-ROC values for DenseNet-EHO suggest its effectiveness in multi-label classification tasks, surpassing the performance of BERT and other methods.

**Table 9:** Metrics AUC-ROC (Benchmark techniques VS MLEn).

Methods/Datasets	Automobiles (%)	Birds (%)	Emotions (%)	Hotel (%)	Medical (%)	Movies (%)	News (%)	Proteins (%)
DenseNet-EHO	0.98	0.99	0.96	0.98	0.97	0.99	0.96	0.96
BERT [52]	0.9	0.9	0.87	0.91	0.89	0.91	0.86	0.88
ML-RBF [30]	0.81	0.83	0.78	0.84	0.8	0.82	0.77	0.79
RAKEL [9]	0.84	0.86	0.81	0.87	0.83	0.85	0.8	0.82
ECC [31]	0.79	0.81	0.76	0.82	0.78	0.8	0.75	0.77
CNN [5]	0.82	0.84	0.79	0.85	0.81	0.83	0.78	0.8
NB [6]	0.8	0.82	0.77	0.83	0.79	0.81	0.76	0.78
LSTM [32]	0.86	0.88	0.83	0.89	0.85	0.87	0.82	0.84
ResNet [14]	0.83	0.85	0.8	0.86	0.82	0.84	0.79	0.81
CapsNet [33]	0.85	0.87	0.82	0.88	0.84	0.86	0.81	0.83
GRU [51]	0.86	0.88	0.83	0.89	0.85	0.87	0.82	0.84

We conclude that the advantages of MLEn over many other sub-learning systems like BERT, ECC and NB support our hypothesis. Integrating learners with machine learning into multilabel learning systems has been shown to have significant advantages over single-label learning systems, particularly in improving system adaptability. DenseNet, in particular, has been successful in this regard due to the ability of its EHO algorithm to diversify among sub-learners. This diversity is a key factor in its success, as it explicitly targets diversity-related goals that were not realized in previous multilabel studies. We then compare the effect of objective functions on results in our proposed technique by comparing MLEn by CNN and Nave Bayes, two distinct types of goal functions.

**Fig 7:**
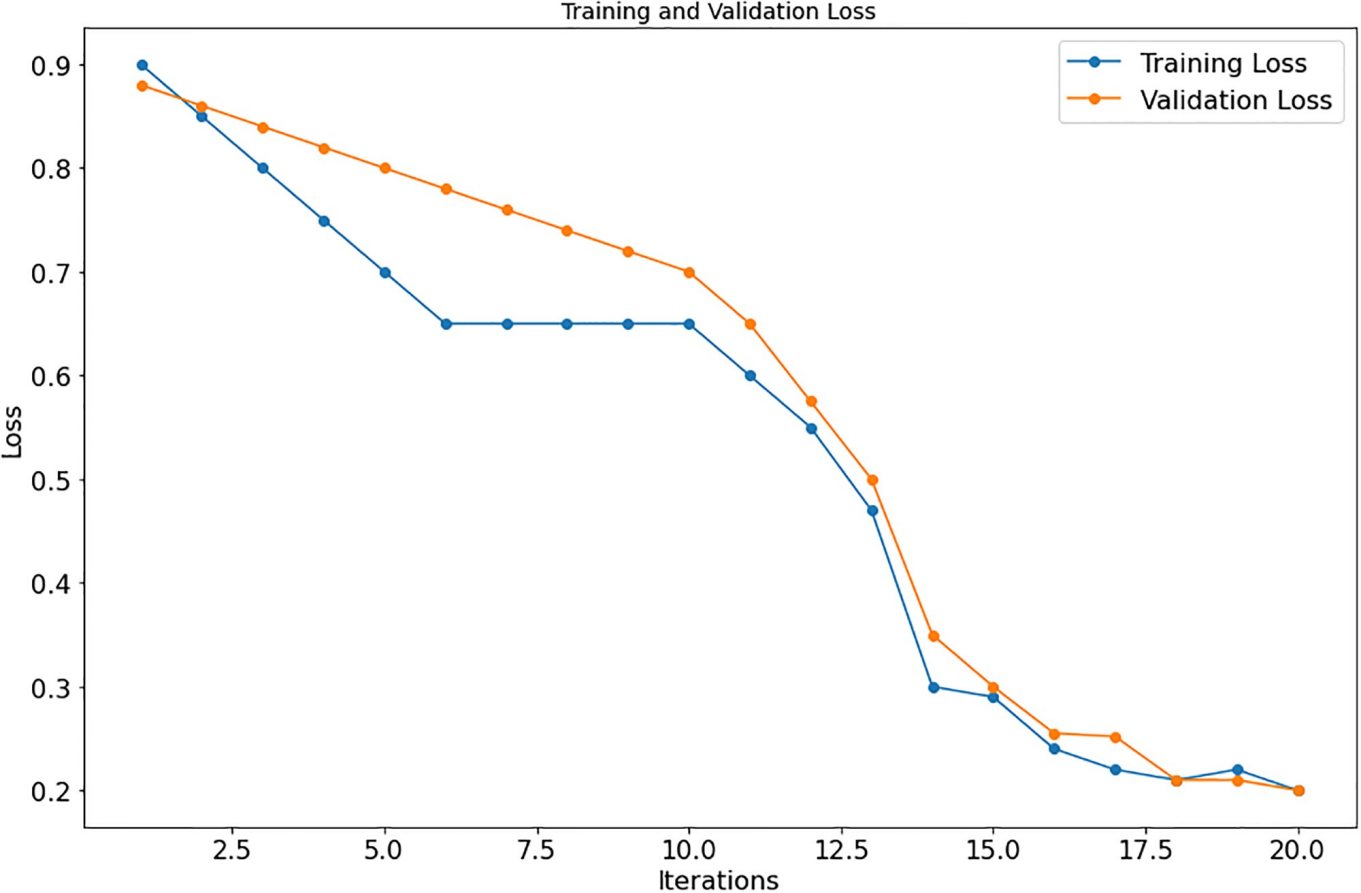
Model loss.

**Fig 8:**
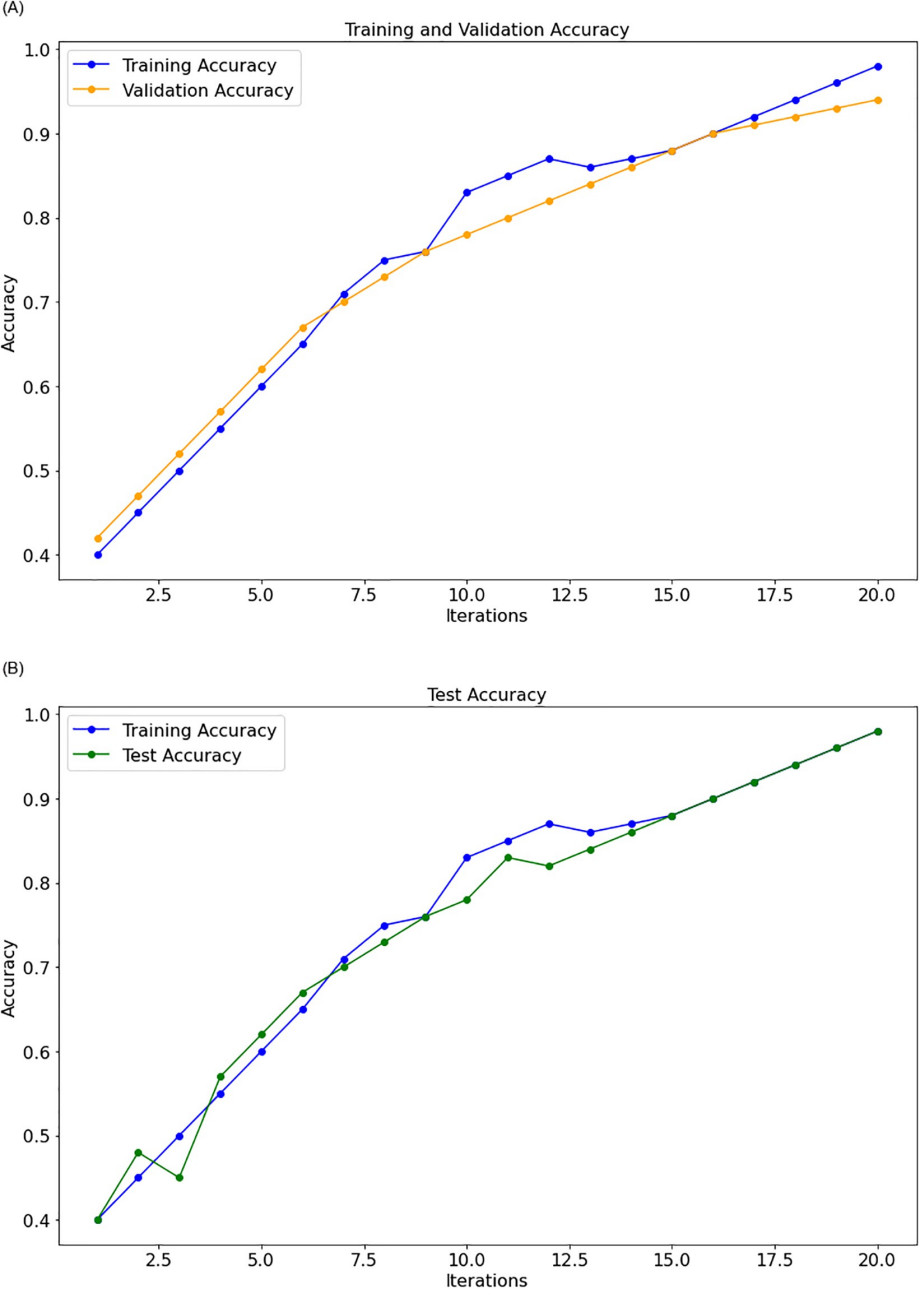
Model accuracy. (**a**) Train-validation, (**b**) Train-Test.

As shown in Tables 3 to 9, the suggested approach consistently yields the best results on each dataset. Figs 7 and 8, respectively, show the model loss, validation, and projected model correctness of the suggested technique. We find that in our suggested strategy, the accuracy of the model increases as the total number of epochs reduces in terms of loss.

**Fig 9:**
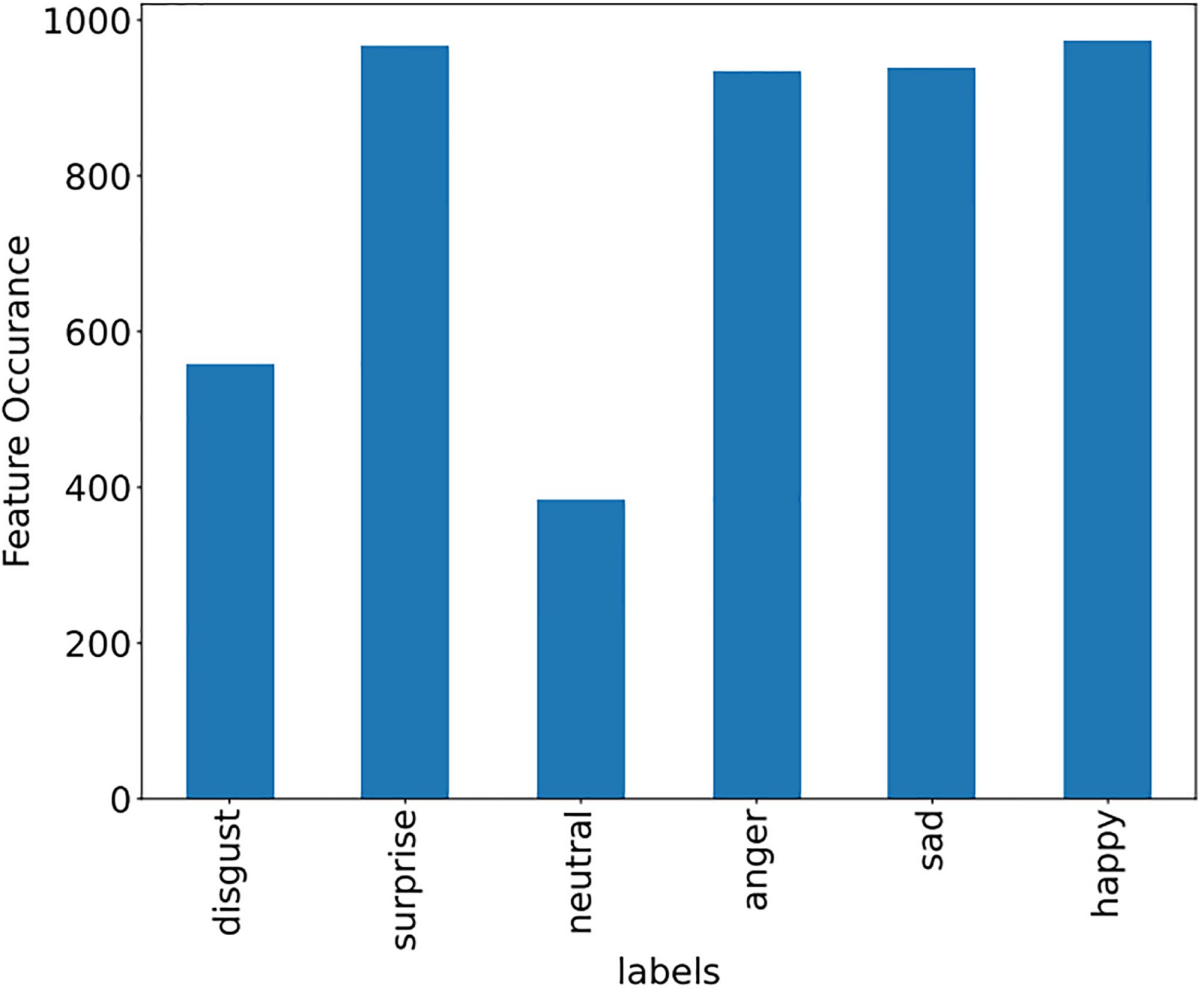
Emotion dataset (Features with frequency).

**Fig 10:**
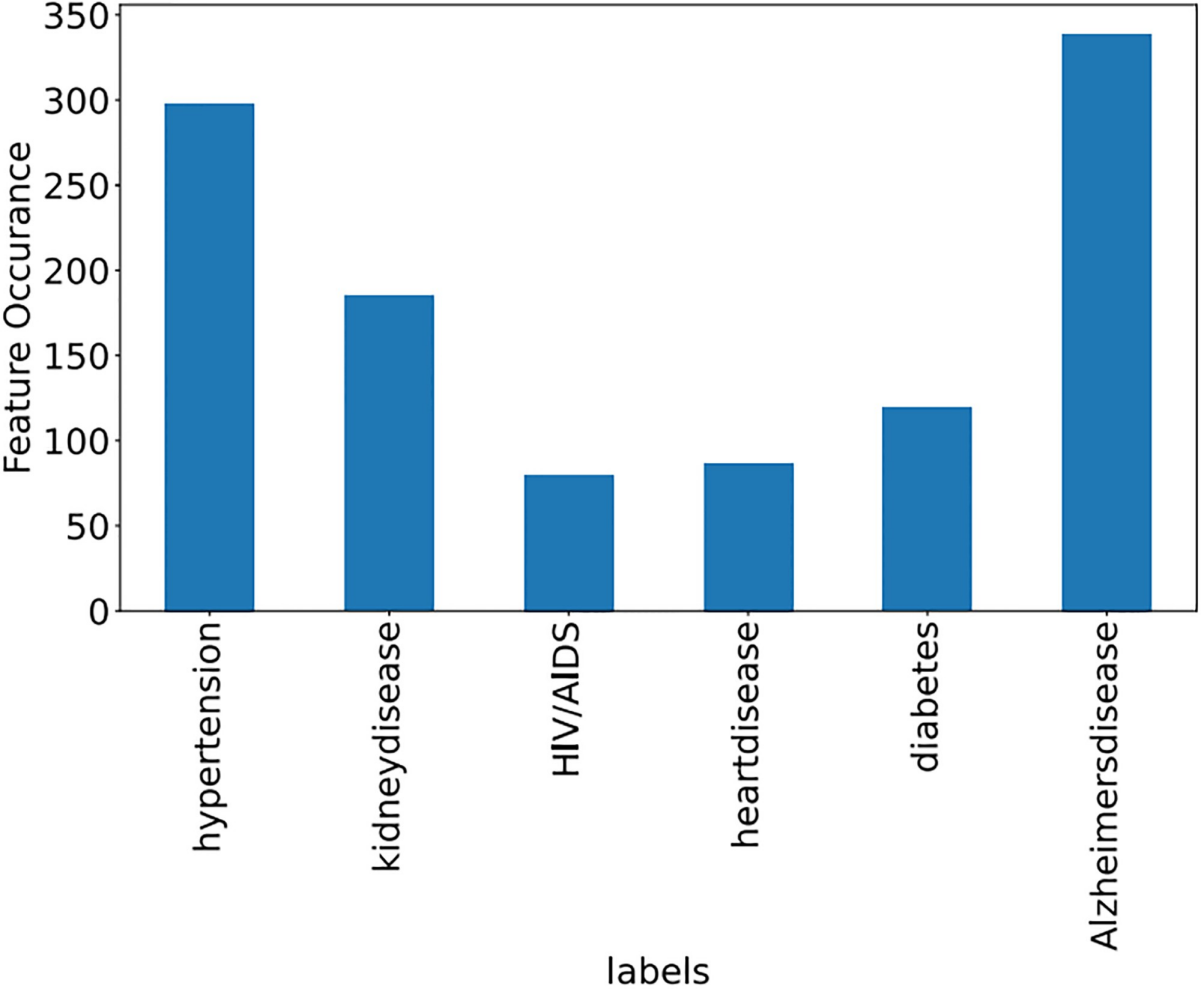
Medical dataset (Features with frequency).

**Fig 11:**
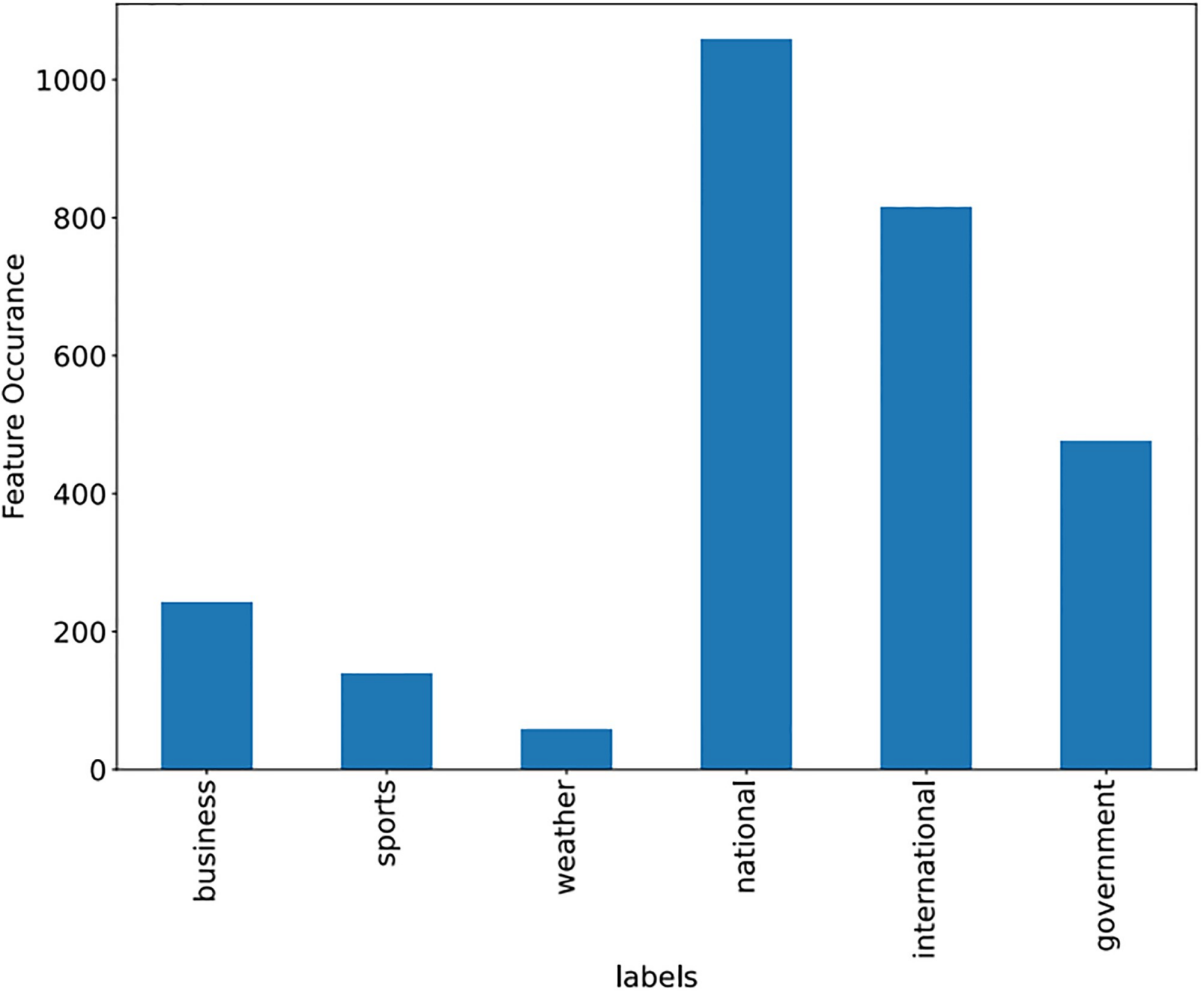
News dataset (Features with frequency).

Some features of the different datasets, along with frequency, are shown in Figs 9–11.

Diverse datasets are evaluated using performance metrics like recall, ROC, accuracy, f-score, and precision.

**Fig 12:**
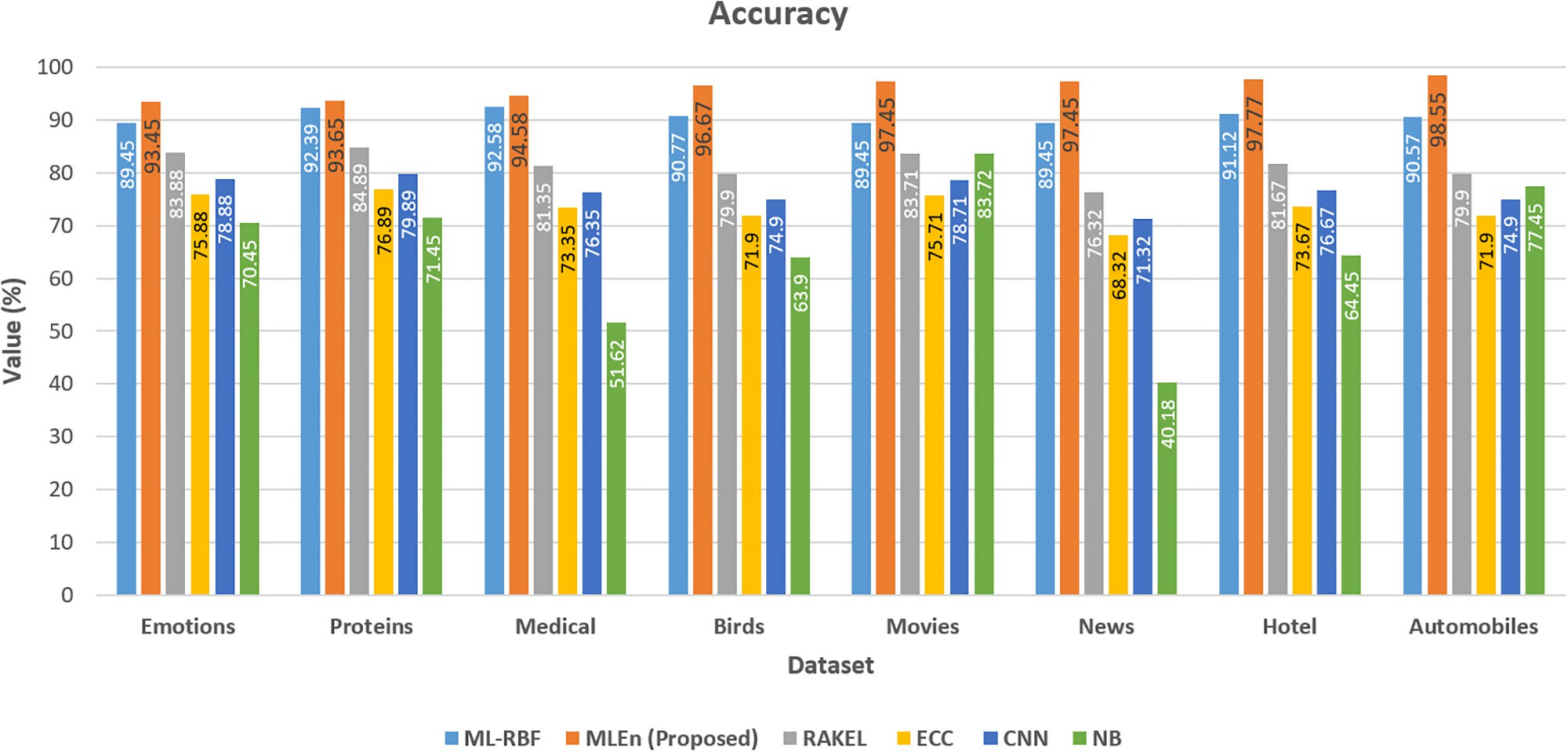
Accuracy of benchmark and proposed method on different datasets.

As it is shown in Fig 12, our proposed ensembler produces an overall better rating on the movie dataset. Despite being a multi-label dataset, the suggested ensembler outperforms even the most challenging approaches. In performance evaluations, as depicted in Fig 12, the recommended ensembler surpasses previous methods. An interesting observation is that the suggested ensembler demonstrates unexpectedly robust results when dealing with multi-labeling data, attributed to its high accuracy.

As the operational output increases in Fig 12, the ML-RBF drops. Fig 12 suggests that MLEan is becoming better. The diverging tendencies of these goals show that they are essentially opposed to one another. Because of the ML-RBF optimization, the anticipated foundation learner labels overlap with labels in the right places. Consequently, these foundational learners all have the same appearance. On the other hand, ECC efficiency allows learners to be as adaptable as possible regarding their training losses. In most cases, the declared objectives are diametrically opposite. The issue presents an opportunity for ML with RBF to successfully balance the two goals through population optimization. In this circumstance, ECC and NB are just assessing the diversity and precision of the fundamental learners, not the ensemble’s overall performance. The ensemble has not declined despite the drop in ML-RBF.

Regardless, the increase in MLEn shows that base learners were expanding, improving group performance. By utilizing a meta-heuristic approach to adjust the values of primary parameters, the performance of the ensembler method has been enhanced, leading to an improvement in the accuracy of multi-labeling. Consequently, multilabel categorization competence is raised by MLEn, which consistently improves a multilabel ensemble’s capacity for generalization.

**Fig 13:**
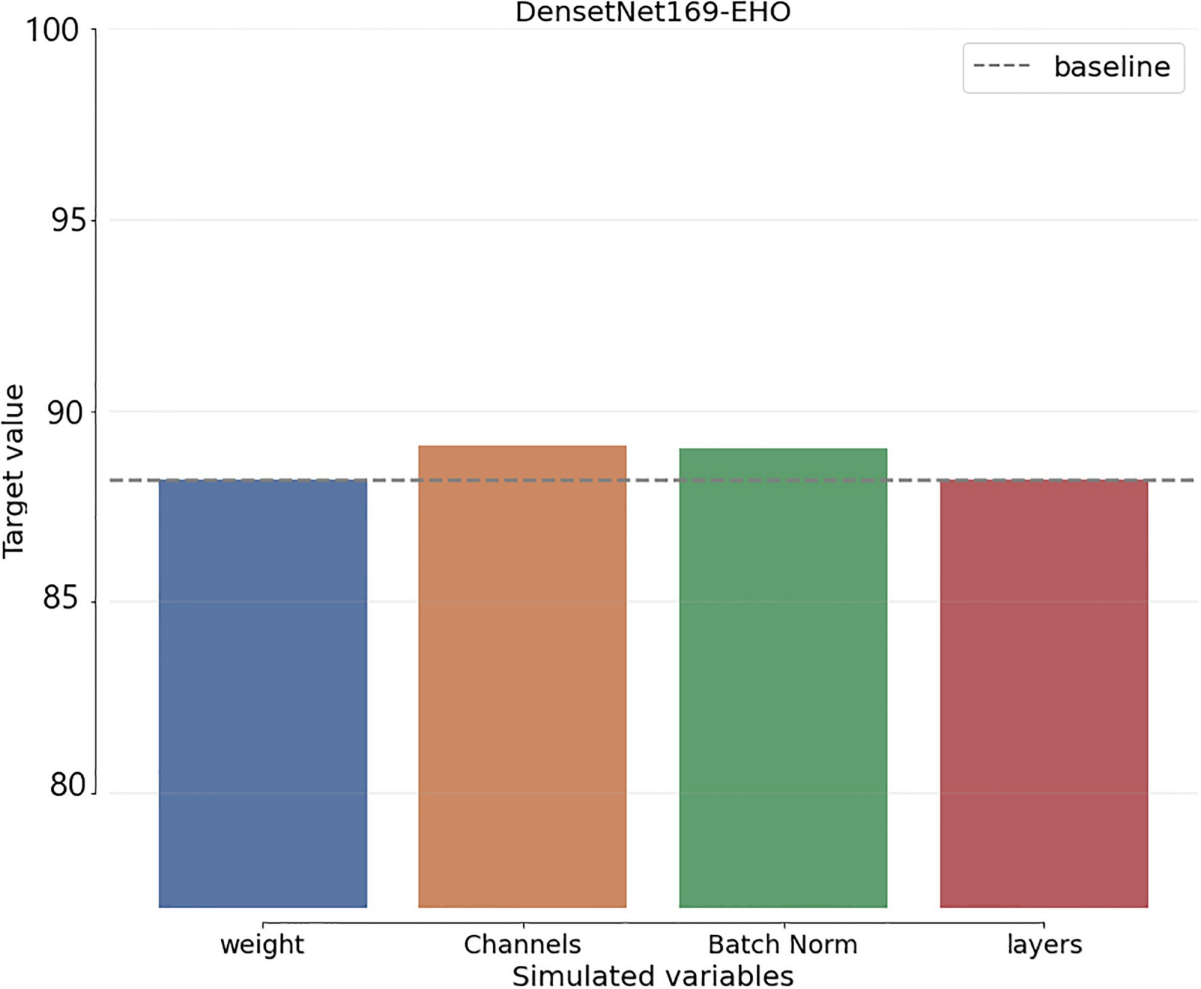
MLEn sensitivity analysis.

To include uncertainty in the benchmark schemes and suggested algorithm decision-making, a Sensitivity Analysis (SA) is employed. Assessing the required modifications for each uncertain element precedes any changes to the initial decision in the SA process. The most accurate projections for the variables in each technique are displayed, together with the degree to which they differ from the initial estimate. As shown in Fig 13, the SA of the proposed approach is computed.

**Fig 14:**
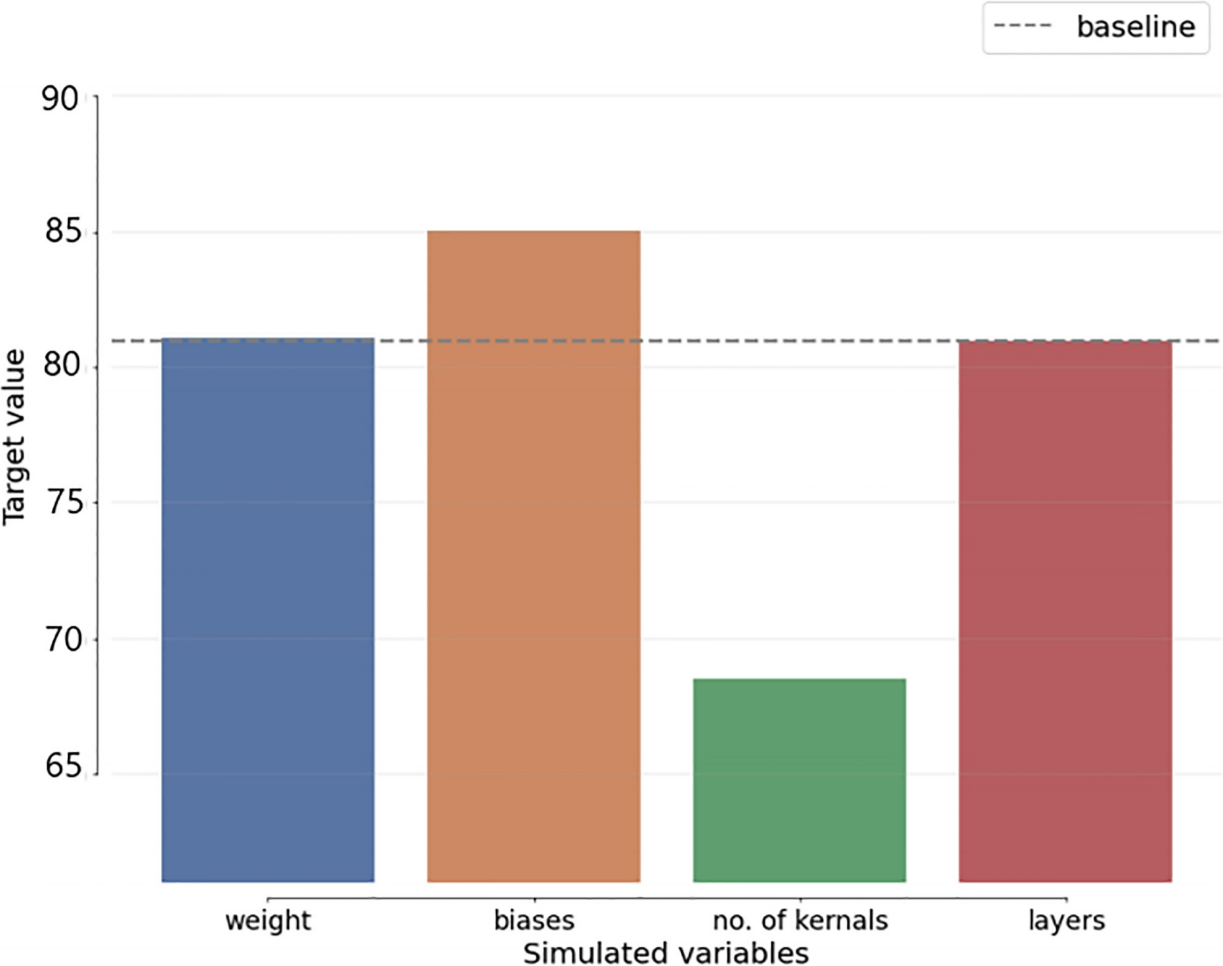
Deep learning CNN model (Sensitivity analysis).

Fig 14 also displays the SA of the most current benchmark techniques. Compared to benchmark algorithms, According to SA comparisons, the baseline-related variables with the lowest variation are the SEn variables.

**Fig 15:**
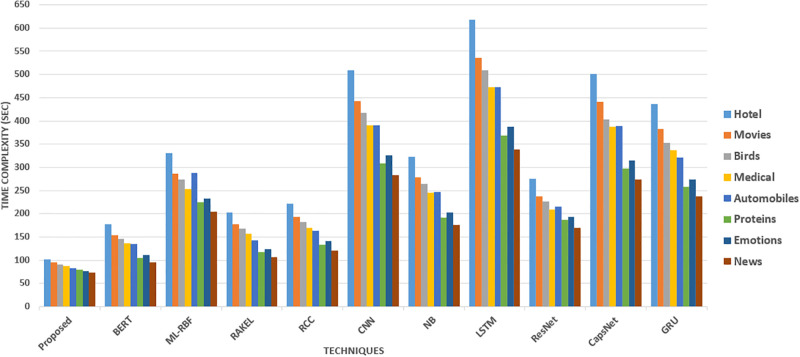
Time complexity of proposed VS existing literature methods.

The Fig 15 represents the time complexity of various methods for different datasets in seconds. The time complexity indicates the time each method requires to perform the given task. Lower time complexity values imply faster execution and better efficiency. Furthermore, DenseNet-EHO stands out with the lowest time complexity, taking less than 75 seconds for all datasets. This indicates that DenseNet-EHO has a significantly faster execution time than other methods. On the other hand, BERT takes around 2.5 to 4.5 minutes, which is relatively higher in comparison.

In the context of ethical considerations in logistics, the discernible differences in time complexity between DenseNet-EHO and BERT become crucial factors influencing the choice of sentiment analysis models. The unique architectural designs and computational demands of DenseNet-EHO and BERT play a pivotal role in determining their applicability in a logistics setting. DenseNet-EHO, a meticulously crafted deep learning model that combines the power of DenseNet with the Elephant Herd Optimization algorithm, exhibits a notable advantage in computational efficiency, resulting in expedited execution. This efficiency aligns with the ethical imperative of optimizing resource utilization in logistical operations. On the other hand, BERT, a renowned transformer-based model, presents itself as a formidable contender with its intricate network structure and substantial computational requirements. The decision to employ BERT would necessitate considering the ethical implications of its heightened computational demands. Furthermore, in the ethical landscape of logistics, the time complexity analysis gains significance. DenseNet-EHO’s lower time complexity can be attributed to its inherent advantages, including an optimized architecture and efficient parameter optimization. The dense connectivity pattern within DenseNet-EHO facilitates streamlined information flow and parameter sharing, minimizing redundant computations and enhancing overall efficiency. This aligns with ethical considerations in logistics, emphasizing the importance of resource efficiency and reduced environmental impact.

**Fig 16:**
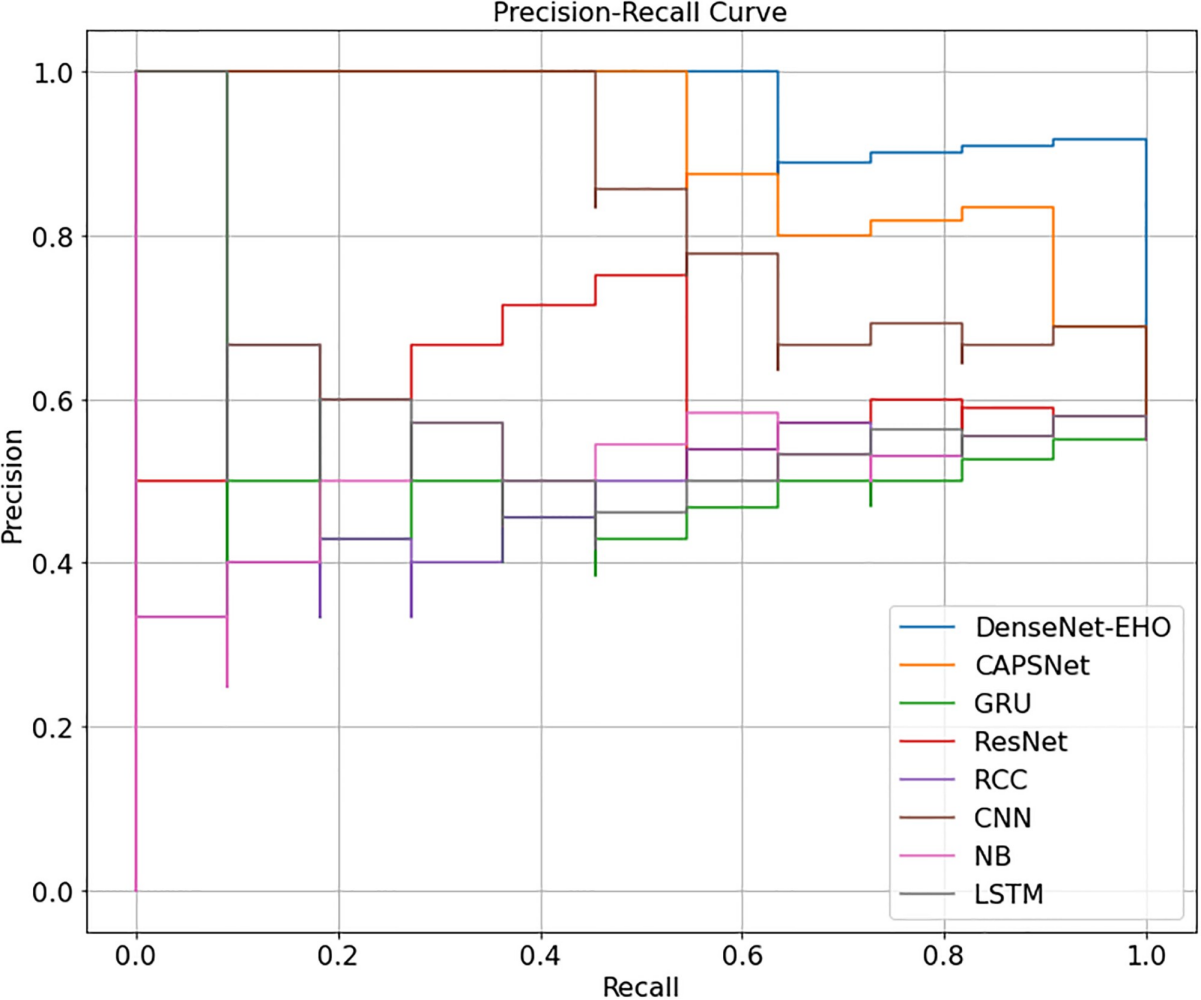
Precision-recall curves for multilabel classification models.

Fig 16 provides a visual snapshot of how various machine learning models, including DenseNet-EHO and BERT, fare in balancing precision and recall when dealing with multilabel classification challenges in the logistics domain. It vividly underscores the exceptional capabilities of the “DenseNet-EHO” model while serving as a benchmark for assessing the performance of other models, some of which may employ random predictions. Essentially, Fig 16 illustrates how different classifiers in the logistics industry trade off precision—the accuracy of positive predictions—and recall—the capacity to record real positive cases. It underscores that the “DenseNet-EHO” model achieves a remarkable precision-recall balance, setting a high ethical standard for comparison.

In short, the disparity in time complexity between DenseNet-EHO and BERT stems from their distinct architectural designs, with DenseNet-EHO showcasing its optimized structure and efficient parameter utilization. These factors, coupled with the inherent benefits of dense connectivity, enable DenseNet-EHO to exhibit swifter execution times—an enticing prospect for researchers seeking computational efficiency and prompt results in their endeavors.

## Conclusion

Our study explores the ethical dimensions of sentiment analysis within the logistics context, introducing MLEn—an innovative approach grounded in an optimization framework. MLEn adopts a multi-objective strategy, effectively addressing the complexities of multi-labeling in logistics. It refines the accuracy of the multi-label learner, assembles ground-based learners, and elevates prediction quality. Utilizing preprocessing techniques, sentiment intensity analysis, and advanced vectorization methods, MLEn establishes a robust foundation for ethical sentiment analysis in logistics. In extensive evaluations across seven datasets, MLEn consistently outperforms established algorithms within the logistics domain, showcasing its relevance and efficiency. Our work has effects for text summarization, extreme multilabeling, and vocabulary training in logistics-specific voice recognition systems, which makes it significant beyond sentiment analysis. The remarkable capabilities of MLEn place our study at the cutting edge of ethical sentiment analysis in logistics, consequently stimulating more research and advancement.

In future, optimizing models like BERT and DenseNet for aspect-based multi-labeling in logistics may require additional investigation. Fine-tuning hyperparameters, refining training processes, and incorporating domain-specific techniques are avenues for achieving higher performance levels. These efforts ensure ethical considerations integral to logistics operations are appropriately addressed, aligning with the evolving landscape of sentiment analysis in logistics.
